# Feature Compression Applications of Genetic Algorithm

**DOI:** 10.3389/fgene.2022.757524

**Published:** 2022-03-08

**Authors:** Meiling Zou, Sirong Jiang, Fang Wang, Long Zhao, Chenji Zhang, Yuting Bao, Yonghao Chen, Zhiqiang Xia

**Affiliations:** ^1^ Institute of Tropical Biosciences and Biotechnology, Chinese Academy of Tropical Agriculture Sciences, Haikou, China; ^2^ Hainan University, Haikou, China; ^3^ Academy of Agriculture and Forestry Sciences, Qinghai University, Xining, China

**Keywords:** fingerprint, DNA molecular markers, SNP, genetic algorithm, feature compression

## Abstract

With the rapid development of molecular breeding technology and many new varieties breeding, a method is urgently needed to identify different varieties accurately and quickly. Using this method can not only help farmers feel convenient and efficient in the normal cultivation and breeding process but also protect the interests of breeders, producers and users. In this study, single nucleotide polymorphism (SNP) data of 533 *Oryza sativa*, 284 *Solanum tuberosum* and 247 *Sus scrofa* and 544 *Manihot esculenta* Crantz were used. The original SNPs were filtered and screened to remove the SNPs with deletion number more than 1% or the homozygous genotype 0/0 and 1/1 number less than 2. The correlation between SNPs were calculated, and the two adjacent SNPs with correlation R^2^ > 0.95 were retained. The genetic algorithm program was developed to convert the genotype format and randomly combine SNPs to calculate a set of a small number of SNPs which could distinguish all varieties in different species as fingerprint data, using Matlab platform. The successful construction of three sets of fingerprints showed that the method developed in this study was effective in animals and plants. The population structure analysis showed that the genetic algorithm could effectively obtain the core SNPs for constructing fingerprints, and the fingerprint was practical and effective. At present, the two-dimensional code of *Manihot esculenta* Crantz fingerprint obtained by this method has been applied to field planting. This study provides a novel idea for the *Oryza sativa*, *Solanum tuberosum*, *Sus scrofa* and *Manihot esculenta* Crantz identification of various species, lays foundation for the cultivation and identification of new varieties, and provides theoretical significance for many other species fingerprints construction.

## Introduction

A DNA molecular marker is a kind of genetic marker based on DNA molecular polymorphism, which directly reflects genetic variation at the DNA level (Muhammad et al., 2018). With the rapid development of molecular biology technology, research on DNA molecular markers is becoming increasingly popular. At present, dozens of different molecular markers have been studied and used, such as restriction fragment length polymorphism (RFLP), random amplified polymorphic DNA (RAPD), amplified fragment length polymorphism (AFLP), and simple sequence repeats (SSR). Single nucleotide polymorphism (SNP), known as a third-generation DNA molecular marker technology, refers to the differences in single nucleotide between different alleles at the same locus. The most common one is the substitution of a single nucleotide, and the deletion or insertion of a single base usually occurs between purine base (A/G) and pyrimidine base (C/T) (Jooyeong et al., 2020). SNP markers play an important role in distinguishing the differences between two genetic materials, so they are also considered the most promising genetic markers. SNP is highly different from the first-generation RFLP and the second-generation SSR markers; this difference is mainly manifested in two aspects. Firstly, the detection tool of SNP is no longer the length change of DNA fragments but directly uses the sequence change as a marker. Secondly, SNP marker analysis completely abandons traditional gel electrophoresis and replaces it with the latest DNA chip technology.

Reduced-representation genome sequencing (RRGS) refers to the use of restriction endonucleases to interrupt genomic DNA to carry out the high-throughput sequencing of specific fragments and obtain a large number of genetic polymorphism marker sequences, thereby fully representing the whole genome information of the target species sequencing strategy. The method is simple in experimental steps and low in cost, and it can obtain genetic polymorphism markers of the whole genome without referring to the genome. It has been widely used in ecology, evolution and genomics. Reduced genome can obtain representative genetic diversity SNPs in the whole genome, so it is widely used to construct population evolution, population structure, population history, genetic mapping and linkage map. At present, the commonly used RRGS technologies includin RAD (Baird et al., 2008)_,_ GBS (Robert et al., 2011)_,_ 2b-RAD (Peterson et al., 2012)_,_ dd-RAD (Shi et al., 2012; Palaiokostas et al., 2016) and AFSM (Xia et al., 2014).

Genetic algorithm (GA) is a search algorithm that simulates natural evolution to explore the optimal solution (Kumar and Ghose, 2009; Wu and Rul’kov, 1993), which belongs to the evolutionary algorithm. It is a computational model of biological evolution based on natural selection and genetic mechanism of Darwin’s theory of biological evolution (Goldberg, 1989). The intersection and penetration between life science and engineering science is the main cause for the emergence of GA, and the highly parallel global random search algorithm used for solving problems is its essence. In the calculation process of GA, the GA cannot directly deal with the parameters in practical problems, and it can only deal with chromosomes expressed in the form of gene strings. Therefore, to use GA, the parameters that optimise the solution of the problem must be transformed into recognisable chromosome form via coding. Aiming at the problem to be solved, an initial population is formed by coding, and the fitness of each individual in the population is calculated. After screening the fitness function, the individuals with high fitness will be operated in the next step. In the operation process, there are three genetic operators, namely, selection operator, crossover operator and mutation operator. Among them, on the basis of the principle that the higher the fitness of the selected individual, the easier it is to be selected and the easier it is to produce the optimal solution, some individuals are selected and hybridised to produce new offspring. Some individuals are selected to mutate to form new offspring and finally form a new population. Re-calculations are conducted according to the process until the fitness of the best individual reaches the set threshold, or when the fitness of the best individual and group reaches the peak, or the number of iterations reaches the set value. The maximum number of iterations is typically adopted as the termination condition. The calculation process of GA is to perform certain operations on individuals depending on the group’s environmental adaptability (adaptability evaluation), so as to realise the evolutionary process of survival of the fittest. From the perspective of optimal search, genetic operation can optimise the solution of the problem by generation and approach the optimal solution.

DNA fingerprinting is a technology based on molecular markers, which has been used to describe the molecular patterns of genotypes. Co-dominant marker (SSR, SNP and SCAR) is the most recommended marker for constructing DNA fingerprinting (Azevedo et al., 2018). This technology is now considered a powerful tool for variety identification, strains and cloning, as well as genetic correlation between parents and offspring (Kopp et al., 2002). The cost of developing DNA molecular markers is becoming low, the speed is becoming fast and the methods are becoming highly convenient and diverse. The DNA fingerprint is gradually improved. Some species have developed from using the first generation of molecular markers RFLP and AFLP to the second generation of molecular markers SSR and then to the third generation of molecular markers SNP, which is beginning to be popular at present. All have constructed fingerprints for a variety of identification or classification processes. With the development of fingerprints, the number of identifiable samples, accuracy and experimental stability have been improved (Chen et al., 2000; Zhan et al., 2012). C.E. McGregor et al. used RAPD (20 primers), ISSR (6 primers), AFLP (2 primers) and SSR (5 primer pairs) to study DNA fingerprints based on PCR in 39 *Solanum tuberosum* varieties (McGregor et al., 2000). A., Zhuk and others used eight SSR markers with the strongest polymorphism to analyse all *Solanum tuberosum* varieties (*Solanum tuberosum* L. subsp. tuberosum) listed in the Latvian plant genetic resources database. The variety fingerprint, genetic distance evaluation and cluster analysis were carried out (Zhuk, 2008). Zhao S J et al. constructed a DNA fingerprint database by SSR markers and analysed the genetic diversity of the main varieties of 27 seedless watermelons in China (Zhao et al., 2013). Li L et al. collected 41 unique wheat varieties in Shandong Province by SSR technology and constructed a DNA fingerprint database (Li, 2013).

How to simply identify new materials and varieties is not only the need of research classification but also an important basis for the protection and utilisation of knowledge output. Therefore, a method that can accurately identify various varieties in different species is urgently needed. Population structure analysis plays a major auxiliary role in variety identification. Cluster analysis is used to observe the genetic relationship of varieties in the population for the further analysis of the origin and variety situation of the varieties. In this study, the SNP data of Gramineae *Oryza sativa*, polyploid *Solanum tuberosum* and mammalian *Sus scrofa* were used. After a series of algorithm screening and population structure analysis, 100 core SNPs were finally selected to construct fingerprints that can distinguish different varieties. These SNPs are expected to be applied to variety specificity and authenticity identification and seedling purity identification, which will provide new technical basis for the further excavation and utilisation of genetic resources of various species and registration and protection of varieties.

## Materials and Methods

### Sample Collection, Sequencing Libraries Preparation and Sequencing

The fresh leaves of 284 *Solanum tuberosum* accessions from the Academy of Agriculture and Forestry Sciences of Qinghai University (E101, N36) were collected, and DNA of all samples was extracted by the modified CTAB method. Through 1% agarose gel electrophoresis detection and concentration measurement, the working solution diluted to 100 ng/μL according to the mother liquor concentration was stored at −20°C. The EcoRI-MspI and EcoRI-HpaII libraries of 284 *Solanum tuberosum* DNA samples were constructed by the AFSM method, and two mixed gene pools of EcoRI-MspI and EcoRI-HpaII were purified by OMEGA’s E.Z.N.A.Cycle Pure Kit. After PCR amplification and monoclonal detection met the requirements, the EcoRI-MspI and EcoRI-HpaII libraries were mixed into a library at a ratio of 1:1 for Hiseq 2,500 sequencing.

### SNP Data Acquisition

For *Solanum tuberosum*, the original Illumina sequencing data were optimised. The artificial data errors were reduced by a Perl script (http://afsmseq.sourceforge.net/), and the total number of sequencing reads was obtained. All the reads were assigned to each individual through the designed tags, and the number of reads of each individual was counted. Optimised sequencing reads were compared with the *Solanum tuberosum* PGSC_DM_v4.03 reference genome by Bowtie2 software, and subsequent SNPs were identified by the two software programs of SAMtools (http://samtools.sourceforge.net/) and VCFtools (http://vcftools.sourceforge.net/). SNP data of *Oryza sativa* and *Sus scrofa* were from the RiceVarMap v2.0 database (http://ricevarmap.ncpgr.cn/) (Chen et al., 2014) and the BIGD database (https://bigd.big.ac.cn/), respectively. SNPs of *Manihot esculenta* Crantz was from our laboratory (Zhang et al., 2018).

### Core SNPs Selection

SNPs were processed in the obtained samples. Genotypes were converted by using online editing tools of awk and sed under the Linux system. The SNPs with the deletion rate over 99%, as well as the homozygous genotype 0/0 and 1/1 number less than 2, were removed. The remaining SNPs were obtained as candidate SNPs. For the candidate SNPs, VCFtools was used to calculate the R^2^ value among SNPs, and the adjacent SNPs with R^2^ > 0.95 were reserved for subsequent calculation.

The read_SNP.m program independently developed in the Matlab platform was used to transform the format of the selected SNPs and pre-process the data, remove all the labelling information in the vcf file and transform the genotype format. The obtained SNPs were used for the calculation of the subsequent GA.

### Application of Genetic Algorithm

**FIGURE 1:**
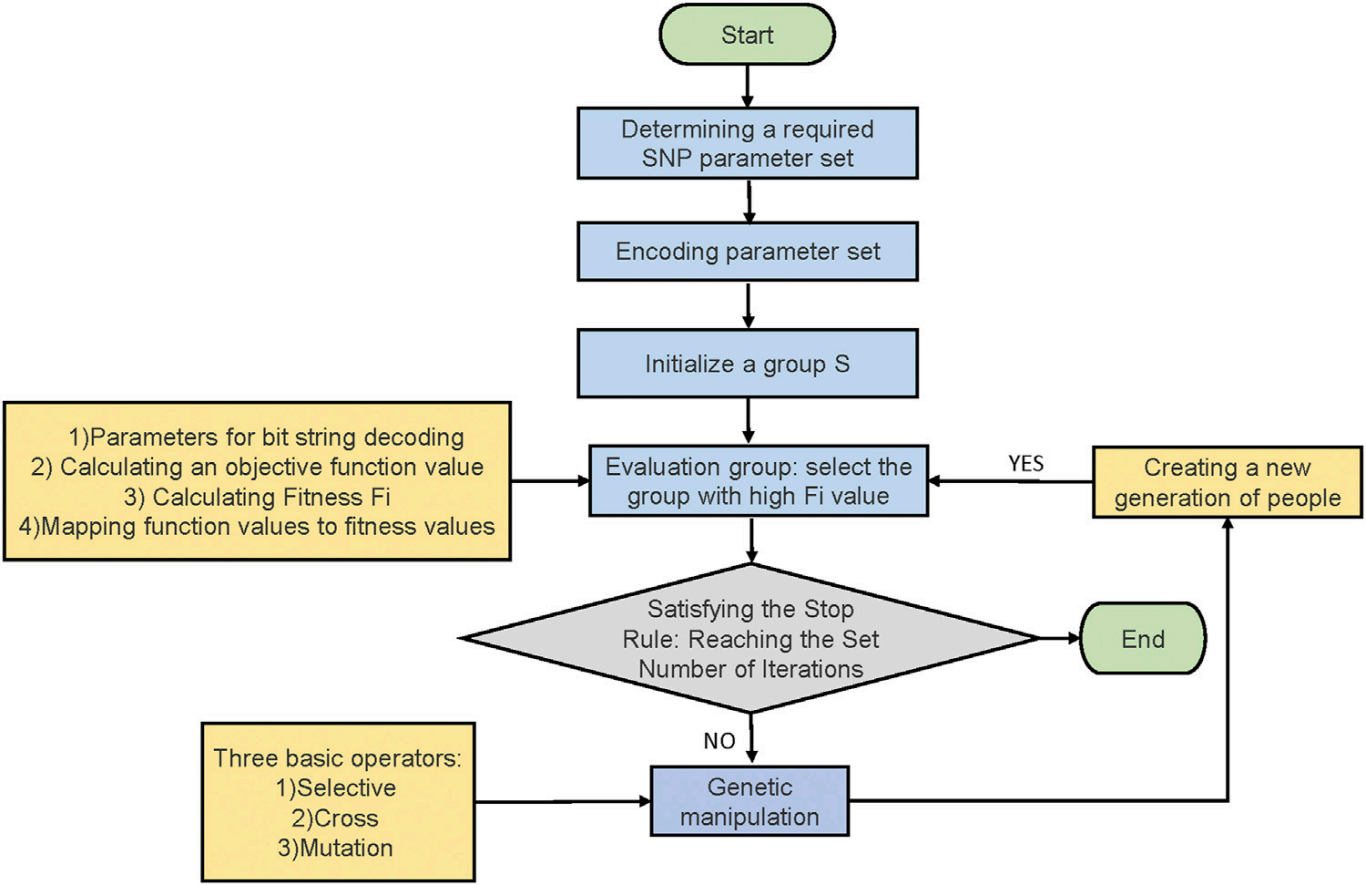
Calculation principle of genetic algorithm.

With the main.m program in Matlab in the same folder, data.mat obtained in the last step was imported to the program automatically. The number of SNPs and the maximum number of iterations in the parameter area were set, then the program was run after deployment (Figure 1). In the running process, the first step was to select the appropriate initial population and the maximum number of iterations and calculate the fitness of the initial population. The formula was as follows:

1) For linear ordering:

$$FitnV\left(pos\right)=2-sp+\frac{2\left(sp-1\right)\left(pos-1\right)}{Nind-1},sp\in \left[1.0,2.0\right].$$ (1)

2) For nonlinear ordering:

$$FintV\left(pos\right)=\frac{Nind\times X^{pos-1}}{\sum_{i=1}^{Nind}X^{i-1}}.$$ (2)

Nind is the number of individuals in a population. sp is the selected differential pressure. pos is a position in an ordered population.

After several iterations, the population number increased. The population with a high fitness was selected for cross-mutation with a new population, then a new population was generated again. The process was repeated until the maximum number of iterations was reached. Then, the procedure was ended. All SNP combinations that can be used in distinguishing *Solanum tuberosum* L. individuals were displayed on the interface. The calculation result was saved in the result file. The detailed information of all the SNP combinations were included in the result file. The SNP combinations were ranked from high to low according to the number of individuals identified. Therefore, a group was selected from several SNP combinations according to the distribution of SNPs on chromosomes for fingerprint construction. The main information obtained from the result file included the ID number of all the SNPs and their positions corresponding to total SNP database. With this information, the core SNP genotype data was collected and arranged.

The specific operation process of genetic algorithm in this study is as follows:

Encoding parameters: the genotypes at SNP loci were transformed into the following four forms by encoding

$$gene=\{\begin{array}{c} 45\;\;\;s=./.\\ 97\;\;\;s=0/0\\ 98\;\;\;s=1/1\\ 104\;\;\;s=0/1 \end{array}$$ (3)

The raw data were obtained from *N* (total number of SNPs) loci of *I* (number of individuals) individuals, and a two-dimensional matrix *S* of N*I (SNP array of all samples) was obtained.

Setting of initial population: We set the population size to be *P*, and each individual randomly selects *C* SNP loci from the *N* SNP loci to form an initial population *G*, which is a two-dimensional matrix of P*C.

Setting of adaptive function: For the individual of each population, we currently have *C* SNP sites randomly selected by it. Assuming that the individual may be any one of the *I* individual samples, data of the corresponding *C* SNP sites in the *I* samples are collected respectively.

$$E\left\{i,j\right\}=\overset{C}{\underset{k=1}{\sum }}S(G\left(i,k\right),j)$$ (4)

1≤i≤P

1≤j≤I

Thus, we can obtain the genotype number coding data e of the randomly selected SNP loci corresponding to each individual in the population.

Next, we find out the unique ones in the *C* genotypes that each individual may have, namely, the valuable SNPs that can be used to distinguish species. The more unique SNPs that an individual may have, the more individuals that it can identify. We believe that the higher the fitness is, and the individual fitness array F of the population can be obtained.

Selection probability:We find the sum *S* of the fitness of *P* individuals. Then the fitness/total fitness of each individual is used to calculate the selection probability of a specific individual in the inheritance

$$S=\overset{P}{\underset{i=1}{\sum }}F\left(i\right)$$ (5)

$$O\left(i\right)=\frac{F\left(i\right)}{S}$$ (6)

1≤i≤P

Cumulative probability: In a roulette-like manner, from 0 to 1, the selection range of each individual is added with the selection probability of the individual from the sum of the selection ranges of all the individuals before, and finally a number in the range of 0–1 is generated to see which range the number falls in, so as to determine which individual is selected finally. for example, the range of the random number *F(j)* required for the selection of the *j*-number individual is as follows

$$\overset{j-1}{\underset{i=1}{\sum }}O\left(i\right)\leq F\left(j\right)\leq \overset{j}{\underset{i=1}{\sum }}O\left(i\right)$$ (7)

Select action: Randomly generating a number of 0–1, selecting individual serial numbers corresponding to two ranges which are closest to the number, and generating a random number *R* which is consistent with uniform distribution on the numbers of 0–1

R=rand(0,1) (8)

Obtaining the serial numbers of the two mated individuals and then mating the two

Interlace operation: It is determined whether or not to perform a crossover operation according to a given crossover probability, and if the crossover operation is performed, a crossover bit *B1* needs to be randomly generated in the range [1,*C*-1]

B1=rand(1,C) (9)

B1(i)∈N+

Taking the crossing position as a dividing line, and carrying out cross exchange on two groups of genes participating in mating, wherein if the error that the SNP locus repeatedly appears in a new genotype after crossing occurs, the SNPs not appearing in the new genotype can be listed from all the *I* SNP loci and sequentially replaced, if the cross operation is not carried out, the offspring keep the original genotype unchanged.

Mutation operation: According to the variation probability, if the mutation operation is performed, 0.3*C mutation bits need to be randomly generated in the [1,*C*] range and counted as *B2* array

B2(i)=rand(1,C) (10)

1≤i≤C

B2(i)∈N+

The mutation site are first emptied, Then listing all the SNP sites which do not currently appear in the variant genotype and sequencing in ascending order, and selecting the SNP sites which do not appear as the variant SNP to be inserted into the variant genotype by taking the value of the variant site as a subscript.

Analog result: As a result, a new population is generated, the previous generation and the new generation should be integrated now, after discarding the SNP with repeated genotypes, the SNP information table of all the existing genotypes is updated, and then the population is eliminated according to the descending order of the fitness of all the genotypic individuals in the current population to ensure that the population number is *P* constant, then the highest fitness, the average fitness and the best SNP individuals of each generation are recorded, and The *X* (maximum number of iterations) generation is cycled in such a way that the last generation is the best combination of SNP loci with the highest fitness, average fitness, and legacy from elimination.

### Evaluate the Number of Core SNPs for Fingerprint Construction

In the calculation process of GA, the number of SNPs was set as 80, 100, 200 and 300, and multiple sets of fingerprints were constructed. The correlation between each matrix of the fingerprint and the original SNP data matrix was calculated in the form of a matrix. The utility of fingerprints composed of different SNPs was evaluated, and fingerprints composed by the best SNP combinations were selected for subsequent verification and analysis.

### Conversion of Genotype Data into Binary Coded Data

Given that SNP molecular markers have dimorphism, the data in the fingerprint database can be converted into binary coded data. Homozygous genotype 0/0 was expressed as 1, homozygous genotype 1/1 was expressed as 2, heterozygous genotype 0/1 was expressed as 0 and deletion base locus was marked as ‘-’. The data of each sample were statistically sorted, so that the genotype data of each sample were integrated to form a unique code, that is, the fingerprint code to distinguish each sample.

### Fingerprint Verification

ADMIXTURE software was used to evaluate the population structure, and each sample in the population was divided into corresponding subgroups. Firstly, the file format required by the software was obtained by using PLINK software (Purcell et al., 2007). Secondly, the value range of subgroup number K was set to 1 to 12, and the appropriate subgroup number K value was selected based on the cross-validation error value obtained from the running result. The software of VCF2DiffMatrix was used to calculate the genetic distances of the core SNP corresponding to the fingerprint library and the original SNP locus after filtration. The distance matrix was obtained and imported into the Neighbour software to calculate the tree file, which was classified according to the K value. Different colours were used to represent different subgroups (https://itol.embl.de/upload.cgi). The results were visualised, and the clustering effect was observed.

The SNPs were visualised by using the CMplot package in R (https://www.r-project.org/), and the SNP distribution variations before and after GA calculation were observed. At the same time, VCFtools were used to calculate the allele frequency of vcf file containing SNP data. The calculated allele frequency was imported into PIC_CALC 0.6 (https://github.com/luansheng/PIC_CALC) software to calculate the PIC value of each SNP locus, count the PIC value of SNP on each chromosome and verify the consistency of data distribution before and after GA calculation. The clustering relationship between the fingerprint database composed of core SNPs and the filtered SNP database was compared. Whether the genetic distance and genetic relationship between each individual are consistent with the obtained results were observed and compared as well (Supplementary Figure S1).

## Results

### SNPs Screening

Screening SNPs according to deletion rate, single variance and linkage relationships between each SNP pairs. The genotype 1/1 was converted to A, 0/0 was converted to B, 0/1 was converted to h and./. was converted to -, which were named as data.mat file. The SNPs with deletion rate of more than 1% and homozygous genotype 0/0 and 1/1 number less than 2 were filtered. A total of 174,819 high-quality SNPs out of the 7,546,976 original SNPs were selected in 533 *Oryza sativa* accessions. The remained high-quality SNPs were imported into Matlab program for subsequent calculation and analysis.

According to the same screening conditions, the data of *Solanum tuberosum* and *Sus scrofa* were filtered. Then the correlation among filtered SNPs were calculated. A total of 1,127 high-quality SNPs out of the 4,786,686 original SNPs were retained in 284 *Solanum tuberosum* accessions, 6,702 high-quality SNPs out of the original 3,667,783 SNPs were retained in 247 *Sus scrofa* accessions and 6,886 high-quality SNPs out of the original 1,641,026 SNPs were retained in 158 *Manihot esculenta* Crantz,respectively. (Table 1).

**TABLE 1:** SNP number after filtration.

Species	*Oryza sativa*	*Solanum tuberosum*	*Sus scrofa*	*Manihot esculenta* crantz
Raw data	7546976	4786686	3667783	1641026
After filtration	174819	1127	6702	6886

### Establishment of Fingerprint of *Oryza sativa*, *Solanum tuberosum* and *Sus scrofa* Based on GA

**FIGURE 2:**
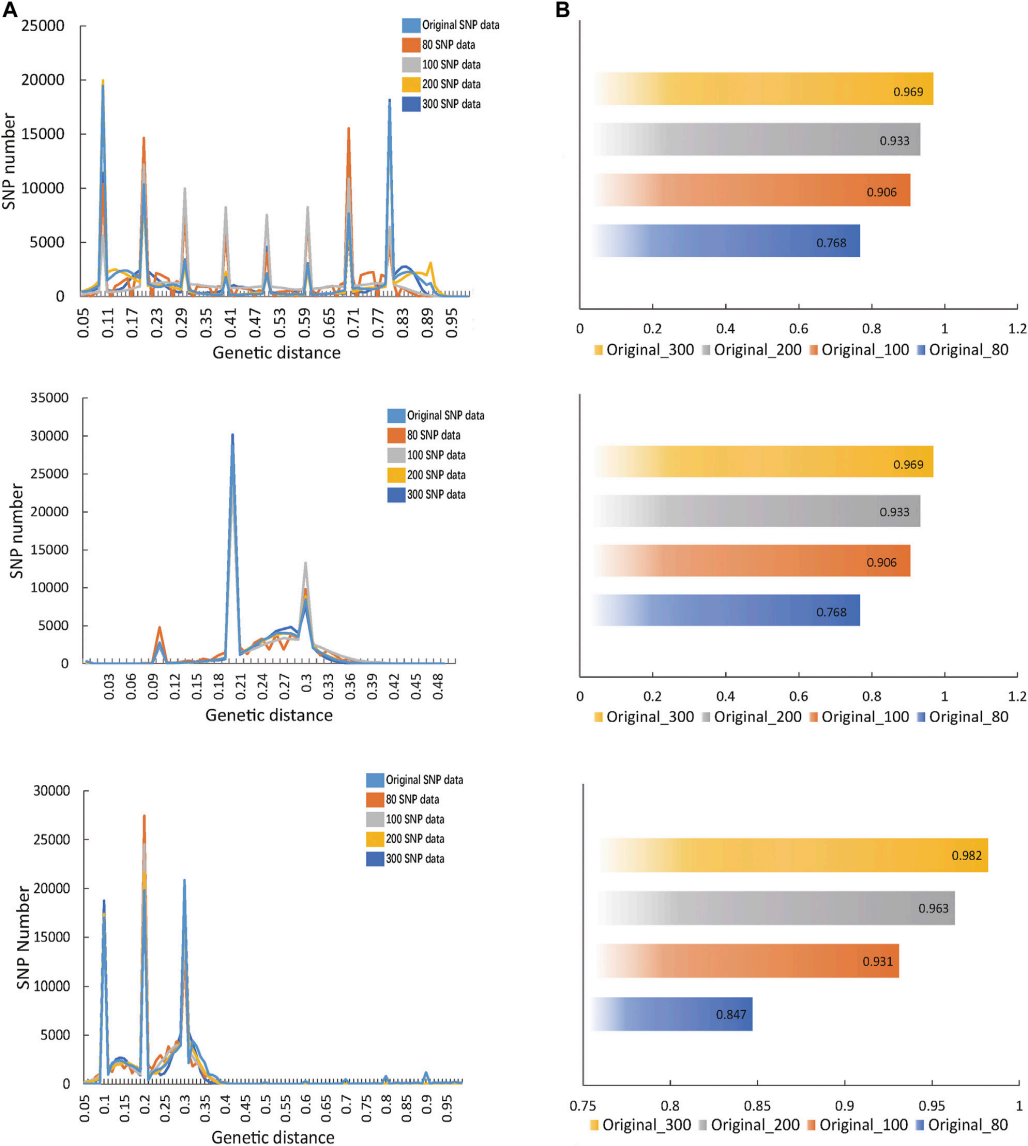
Calculation of the optimal number of SNPs in fingerprint. **(A)** The fitting diagram of comparing fingerprints composed of different SNPs with original SNP data. Dark blue represents the graph of original SNP data, orange represents the fingerprint curves of 80 SNPs, grey indicates the fingerprint curves of 100 SNPs, light blue shows the fingerprint curves of 200 SNPs and yellow illustrates the fingerprint curves of 300 SNPs. **(B)** The correlation value between each fingerprint and the original SNP data.

Through GA calculation, the fingerprint matrix constructed by 80, 100, 200 and 300 SNPs was obtained. By calculating the correlation between the fingerprint matrix and original SNP matrix, the fitting degree with original data became high with the increase in the SNP number. However, when there were 100 SNPs, it had been basically fitted with the original data (Figure 2A). From a numerical perspective, compared with the fingerprint composed of 80 SNPs, the correlation index between the fingerprint composed of 100 SNPs and the original data demonstrated a sudden increase (Figure 2B). Therefore, the better fingerprint data of 533 *Oryza sativa* populations were composed of 100 SNPs, and the fingerprint composed of 100 core SNPs was finally retained. In the same way, the fingerprints composed of 80, 100, 200, and 300 SNPs calculated from *Oryza sativa* and *Sus scrofa* were compared with the original data, and the fingerprints composed of 100 SNPs had a good fitting degree with the original data.(Supplementary Tables S2–S4).

The genotypic values of core SNPs of 533 *Oryza sativa* varieties, 284 *Solanum tuberosum* varieties and 247 *Sus scrofa* varieties were transformed into binary coding data 0, 1 and 2, respectively. The fingerprint database of all *Oryza sativa*, *Solanum tuberosum* and *Sus scrofa* varieties was obtained (Supplementary Tables S5–S7). The fingerprint codes obtained were converted into two-dimensional code format, which could better and more conveniently distinguish and identify different variety populations, it has been used in *Manihot esculenta* Crantz. (Supplementary Table S8).

### Population Structure Analysis of *Oryza sativa*, *Solanum tuberosum* and *Sus scrofa* Populations

**FIGURE 3:**
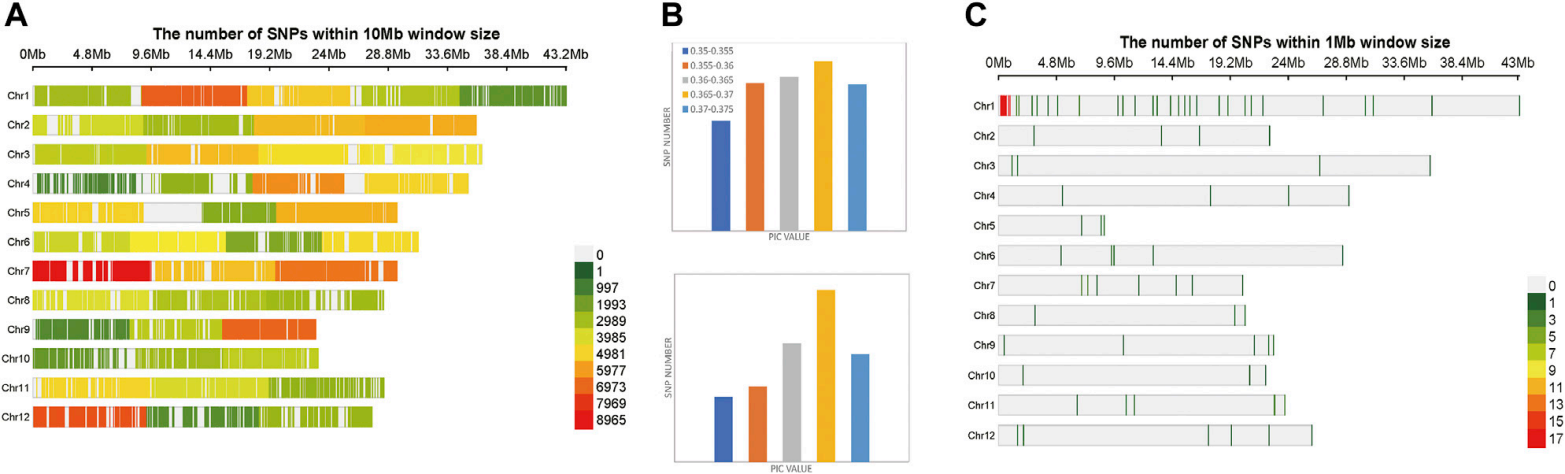
Variation statistics of SNPs in *Oryza sativa* before and after genetic algorithm calculation. **(A)** The density distribution of original SNPs of *Oryza sativa*. **(B)** The PIC value statistics before and after genetic algorithm calculation. **(C)** The density distribution of 100 SNPs of *Oryza sativa* fingerprint.

A total of 174,798 SNPs were obtained, which were evenly distributed on 12 chromosomes of *Oryza sativa,* after two-step filtration. The distribution, number and density of SNPs on 12 chromosomes of *Oryza sativa* were showed in Figure 3A. At the whole genome level of *Oryza sativa*, the average polymorphism information content was 0.363. The polymorphism information content of most SNPs was between 0.365 and 0.37, and the polymorphism information content of very few SNPs was between 0.35 and 0.355. These values indicated that the polymorphism information content of *Oryza sativa* SNPs was high and distributed evenly. After selection and GA calculation, a fingerprint map constructed by 100 core SNPs was obtained. The SNPs were evenly distributed on 12 chromosomes of *Oryza sativa*. Figure 3C shows the distribution, number and density of 100 SNPs of fingerprint on 12 chromosomes of *Oryza sativa*. The selected core SNPs were at the whole genome level of *Oryza sativa*, and the average polymorphism information content was 0.364. The PIC values of 100 SNPs were mostly distributed between 0.365 and 0.37, with a total number of 32. The data showed that the obtained 100 core SNPs calculated by GA were consistent with the original filtered data.

The *Oryza sativa* population structure analysis of all the 174,798 high-quality SNPs was carried out by admixture software. The maximum cluster subgroup number (K) was estimated to be a certain value between 2 and 14, and the cross-validation error rate (CV error) under each K value was calculated. When K was from 1 to 9, CV error decreased rapidly. When K was greater than 9, CV error increased gradually and tended to be flat (Figure 6A). Therefore, when K was equal to 9, CV error was the smallest, indicating that K = 9 was the most suitable, that is, the whole *Oryza sativa* population was divided into nine subgroups, namely, subgroup 1 to subgroup 9. In R, according to K = 9, the *Oryza sativa* population was divided into nine subgroups. K-means clustering analysis was carried out on the filtered original SNP database and the obtained fingerprint database, and the K-value matrix was obtained. Genetic distance was calculated by VCF2DiffMatrix software, which was imported into the Neighbour software to obtain a tree file. The nine subgroups were classified and expressed by nine different colours, and the evolutionary tree was visualised in iTOL to obtain clustering results. The clustering results of 100 SNP fingerprint database were basically similar to those of all 174,798 SNPs, indicating that the use of 100 SNPs for fingerprint construction was effective (Figure 6B).

**FIGURE 4:**
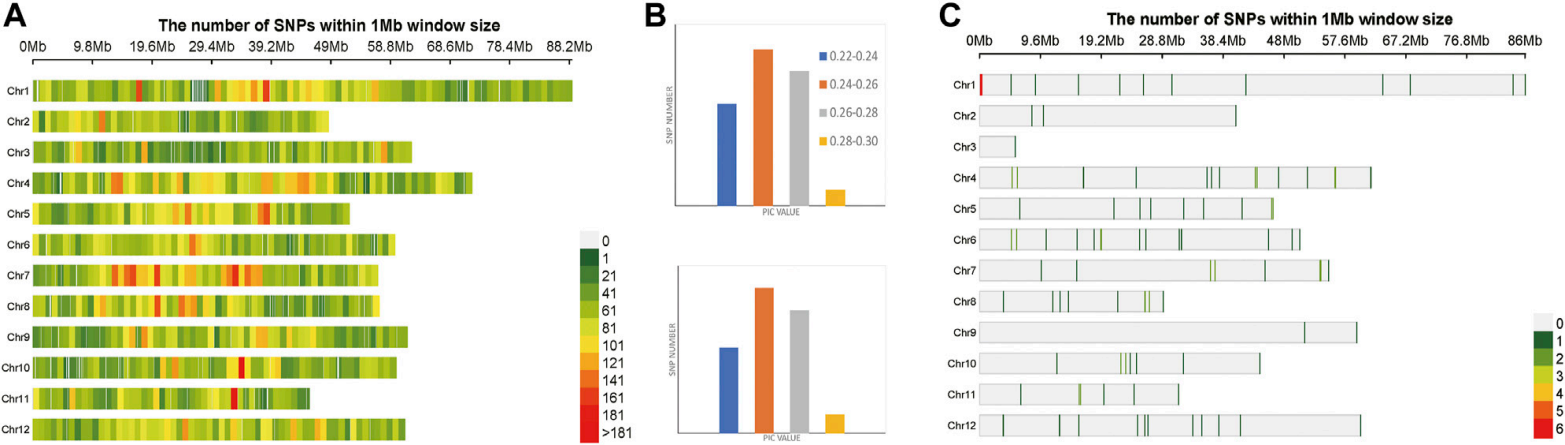
Variation statistics of SNPs in *Solanum tuberosum* before and after genetic algorithm calculation. **(A)** The density distribution of original SNPs of *Solanum tuberosum*. **(B)** The PIC value statistics before and after genetic algorithm calculation. **(C)** The density distribution of 100 SNPs of *Solanum tuberosum* fingerprint.

A total of 1,127 SNPs were obtained, after two-step filtration, which were distributed evenly on 12 chromosomes of *Solanum tuberosum*. Figure 4A shows the distribution, number and density of SNPs on 12 chromosomes of *Solanum tuberosum*. The number of mutations on each chromosome ranged from 38 to 135, with an average of 94; the largest number of SNPs 135) was on chromosome 1. By contrast, there were 38 SNPs, the least number of SNPs, on chromosome 2. At the whole genome level of *Solanum tuberosum*, the average polymorphism information content was 0.249. The polymorphism information content of most SNPs was between 0.24 and 0.26, and the polymorphism information content of very few SNPs was between 0.2 and 0.22. These values indicated that the polymorphism information content of *Solanum tuberosum* SNPs was relatively even. After selection and GA calculation, a fingerprint constructed by 100 core SNPs was obtained, and the SNPs were distributed evenly on 12 chromosomes of *Solanum tuberosum*. Figure 4C shows the distribution, number and density of 100 SNPs of fingerprint on 12 chromosomes of *Solanum tuberosum*. The statistical results showed that the number of SNPs on chromosome 1 was the largest (36). However, the one with the least number of SNPs occurred on chromosome 5, followed with chromosome 2. These observations showed that the distribution of sites was relatively even and consistent with the distribution of the original calculated data filtered. The selected core locus was at the whole genome level of *Solanum tuberosum*, and the average polymorphism information content was 0.255. The PIC values of 100 SNPs were mostly distributed between 0.24 and 0.26, with a total of number of 39. The average polymorphism information content of the SNPs on each chromosome was between 0.241 and 0.268. The highest average polymorphism information content was on chromosome 9, reaching 0.268, while the lowest was on chromosome 2, which was only 0.2411. After the calculation of GA, the obtained 100 core SNPs were consistent with the original filtered data.

The admixture software was used to carry out the *Solanum tuberosum* population structure analysis of all 1,127 high-quality SNPs. The maximum number of cluster subsets (K) was inferred to be a certain value between 1 and 10, and the CV error under each K value was calculated. When K was from 1 to 7, CV error decreased rapidly. After K was greater than 7, CV error increased gradually and tended to be flat (Figure 6A). Therefore, when K was equal to 7, CV error was the smallest, indicating that K = 7 was the most suitable, that is, the whole *Solanum tuberosum* population was divided into seven subgroups, namely, subgroup 1 to subgroup 7. In R, according to K = 7, the *Solanum tuberosum* population was divided into seven subgroups. K-means clustering analysis was carried out on the filtered original SNP database and the obtained fingerprint database, and the K-value matrix was obtained. Genetic distance was calculated by VCF2DiffMatrix software, which was imported into the Neighbour software to obtain a tree file. Seven subgroups were classified and expressed by seven different colours, and the evolutionary tree was visualised in iTOL to obtain clustering results. The clustering results of 100 SNP fingerprint database were basically similar to those of all 1,127 SNPs, indicating that the use of 100 SNPs for fingerprint construction was effective (Figure 6B).

**FIGURE 5:**
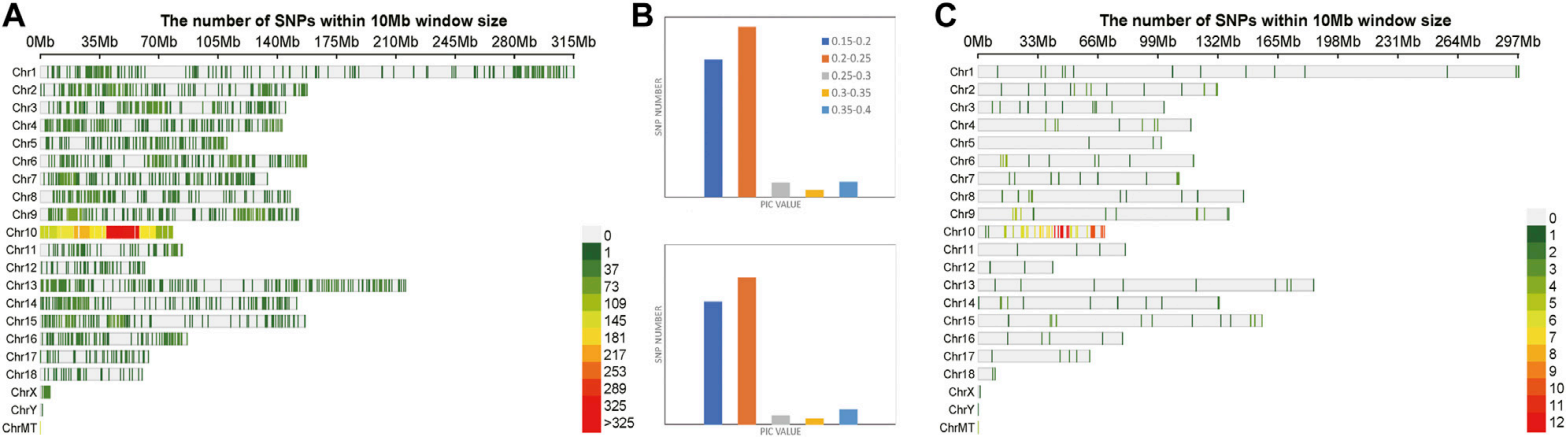
Variation statistics of SNPs in *Sus scrofa* before and after genetic algorithm calculation. **(A)** The density distribution of original SNPs of *Sus scrofa*. **(B)** The PIC value statistics before and after genetic algorithm calculation. **(C)** The density distribution of 100 SNPs of *Sus scrofa* fingerprint.

A total of 6,702 SNPs were obtained after two-step filtration, which were distributed evenly on 21 chromosomes of *Sus scrofa*. Figure 5A shows the distribution, number and density of SNPs on 21 chromosomes of *Sus scrofa*. At the whole genome level of *Sus scrofa*, the average polymorphism information content was 0.217. The polymorphism information content of most SNPs was between 0.2 and 0.25, and the polymorphism information content of very few SNPs was between 0.3 and 0.35. After selection and GA calculation, a fingerprint constructed by 100 core SNPs was obtained, and the SNPs were distributed relatively evenly on 21 chromosomes of *Sus scrofa*. Figure 5C shows the distribution, number and density of 100 SNPs of fingerprint on 21 chromosomes of *Sus scrofa*. After the GA calculation, the distributions of SNPs on each chromosome were relatively even, which were consistent with the distributions of the filtered original calculation data. The selected core locus was at the whole genome level of *Sus scrofa*, and the average polymorphism information content was 0.216. The PIC values of 100 SNPs were mostly distributed between 0.2 and 0.25, with a total of number of 49. After the calculation of GA, the obtained 100 core SNPs were consistent with the original filtered data.

**FIGURE 6:**
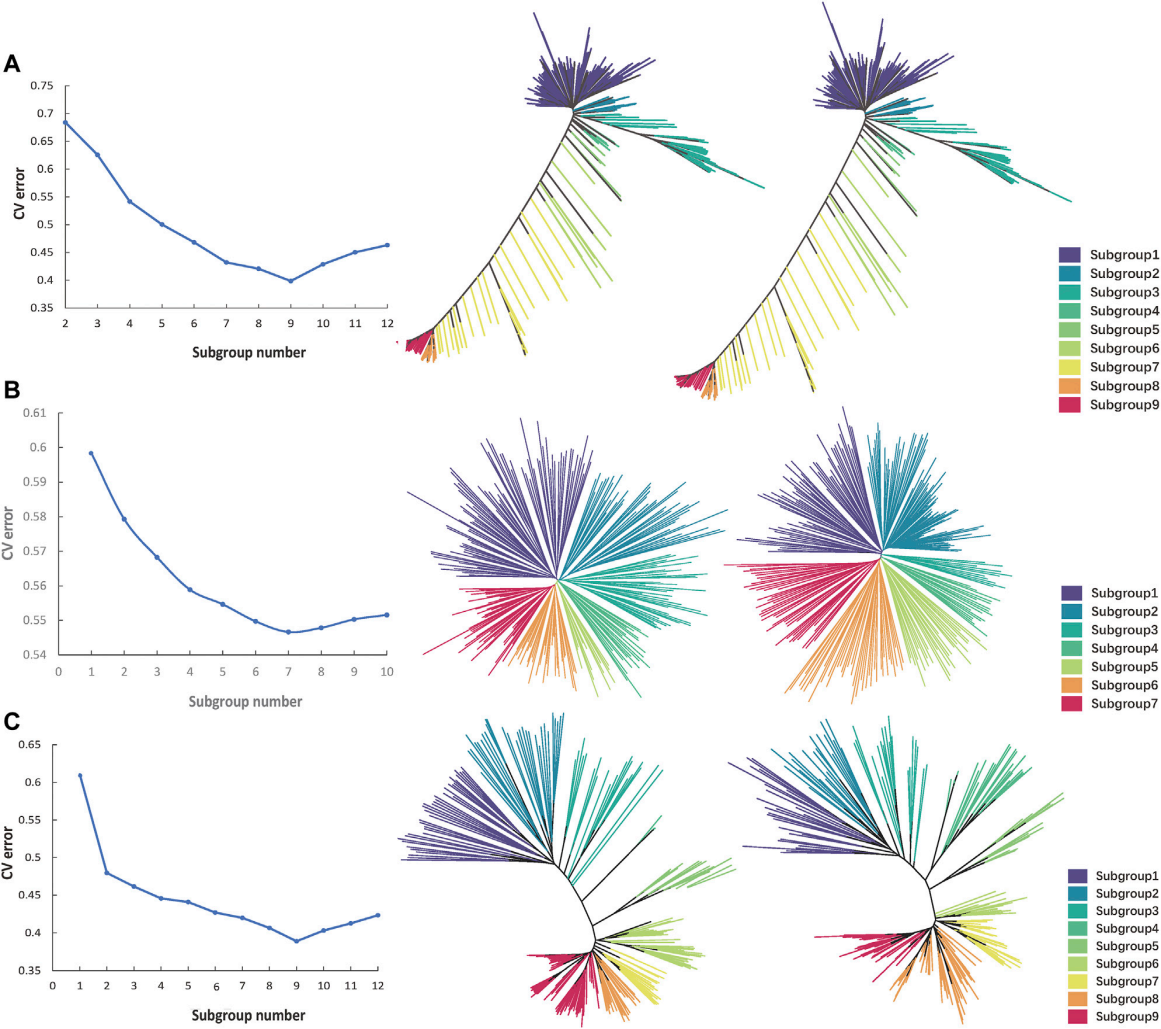
Cluster comparison of each species before and after genetic algorithm calculation. **(A)** The CV error value statistics and the Nj cluster tree of 533 *Oryza sativa* populations. The left one shows the clustering of original data, and the right one shows the clustering of fingerprint data. Different colours represent different subgroups. **(B)**. The CV error value statistics and the Nj cluster tree of 284 *Solanum tuberosum* populations. **(C)**. The CV error value statistics and the Nj cluster tree of 247 *Sus scrofa* populations.

The admixture software was used to carry out the *Sus scrofa* population structure analysis of all 6,702 high-quality SNPs. The maximum number of cluster subsets (K) was inferred to be a certain value between 1 and 12, and the CV error under each K value was calculated. When K was from 1 to 9, CV error decreased rapidly. When K was greater than 9, CV error increased gradually and tended to be flat (Figure 6A). Therefore, when K was equal to 9, CV error was the smallest, indicating that K = 9 was the most suitable, that is, the whole *Sus scrofa* population was divided into nine subgroups, namely, subgroup 1 to subgroup 9. In R, according to K = 9, the *Sus scrofa* population was divided into nine subgroups. K-means clustering analysis was carried out on the filtered original SNP database and the obtained fingerprint database, and the K-value matrix was obtained. Genetic distance was calculated by VCF2DiffMatrix software, which was imported into the Neighbour software to obtain a tree file. The nine subgroups were classified and expressed by nine different colours, and the evolutionary tree was visualised in iTOL to obtain clustering results. The clustering results of 100 SNP fingerprint database were basically similar to those of all 6,702 SNPs, indicating that the use of 100 SNPs for fingerprint construction was effective (Figure 6B).

### Application of Core SNP in *Solanum tuberosum* Variety Identification

To determine the genetic relationship among *Solanum tuberosum* varieties in detail and whether the fingerprint is true and effective, the genetic distances among 284 individuals were calculated by evaluating and statistically utilising the DNA-SNP database calculated by GA. The average genetic distance among all varieties was 0.15359, ranging from 0.05 to 0.415. When the genetic distance was the minimum value of 0.1, there were two groups of individuals (R3R4 and CIP09-29 and KW-59 and KW-11). Every two individuals in these two groups of individuals were similar in origin, and they all came from CIP. There were two groups of individuals (Sebago-1 and Ziyun and CIP09-29 and zhongshu18) when the genetic distance was the maximum value of 0.65. These two groups of individuals had different origins. The origin of Sebago-1 was unknown, while Ziyun came from China, CIP09-29 came from CIP and zhongshu18 came from China. We compared the genotypes of 100 SNP loci and found that the number of different loci between R3R4 and CIP09-29 is 28, the number of different loci between KW-59 and KW-11 is 25, and that between Sebago-1 and Ziyun The number of different loci is 38, and the number of different loci between CIP09-29 and zhongshu18 is 42, which is significantly more than the previous two groups. These findings showed that the fingerprint could show the origin and genetic relationship of the *Solanum tuberosum* population, and the results were reliable and available.

## Discussion

### Construction of Fingerprint Based on SNP Genotype Data

DNA fingerprinting is an applied genetic germplasm analysis method established with the development of molecular biology. There are different traits and DNA sequences among various species and varieties, and varieties can be distinguished according to these differences (Sharon et al., 1992).

At the end of the 1970s, the technology of studying the differences in DNA among different individuals was proposed and applied. The differences in DNA molecules can directly reflect the differences between individuals and are not affected by the external environment. Therefore, it gradually replaces the previous method of observing external phenotypic differences with the naked eyes to distinguish individuals. With the development of molecular biology, various DNA molecular marker technologies have been widely studied and applied, from RFLP, AFLP to later developed SNP. These molecular markers have played a critical role in the analysis and research of fingerprint constructions.

However, the previous molecular markers have some shortcomings. Relatively speaking, RFLP technology is cumbersome to operate and has low polymorphic information content, which has limitations in individual recognition. The RAPD technique has poor repeatability and unstable results, so it can obtain reliable results only by optimising the reaction conditions repeatedly. AFLP technology has high requirements on genome purity and reaction conditions. Compared with the previous ones, SSR technology has the advantages of high polymorphism detection rate, large information content, simple experimental operation and stable and reliable results, but it also has the disadvantages of low detection efficiency and low repeatability (Wang, 2006). Li et al. (Li et al., 2017)developed a novel method for SSR genotyping, named as AmpSeq-SSR, which combines multiplexing polymerase chain reaction (PCR),targeted deep sequencing and comprehensive analysis.The identification fingerprint of that rice variety construct by 3,105 ssr markers is obtain by the method, which offer much greater discriminative power than the 48 SSRs commonly used for rice. As the third-generation molecular marker, SNP has unique advantages, which solves the unrepeatability of SSR markers well. It has the following advantages. Firstly, the SNPs are abundant and almost all over the whole genome. Secondly, it is rich in polymorphism and has higher genetic stability than SSR. In addition, SNP detection methods are becoming increasingly simple and efficient and have excellent application prospects.

Traditionally, SSR is used to evaluate the genetic characteristics of species (Mba et al., 2001; Hurtado et al., 2008). However, SSR rarely produces multi-allele markers in *Solanum tuberosum*, which makes the study of genetic identification or genetic variation time-consuming and laborious. SNP is a double allele genotype marker, which is more abundant than SSR, easy to be automated, highly repetitive and can be shared among laboratories (Primmer et al., 2002; Floro et al., 2018). Therefore, SNP was selected to complete this study.

### Construction of Fingerprint by GA

In the field of artificial intelligence, finding the optimal solution or approximate optimal solution in a very large and complex space is a key problem that has always existed. For NP-hard problems (Komusiewicz et al., 2014), if an improper search method is used, problems such as combinatorial explosion may occur. Therefore, researchers in related fields have been devoted to find a general search algorithm. Pre-exact algorithm and intelligent optimisation algorithm are common solutions to function optimisation problems. Accurate algorithms are time-consuming and suitable for small-scale problems. Intelligent optimisation algorithms mainly include simulated annealing algorithm proposed by reference (Holland, 2006) in 1953, GA proposed by reference (Glover, 1986) in 1973, tabu algorithm proposed by reference (Colorni et al., 1992) in 1986, ant colony algorithm proposed by reference (Hopfield, 1982) in 1992 and neural network method used by reference (Jungnickel, 2008) in an innovative way.

Genetic algorithm, Particle Swarm optimization, and Differential Evolution Algorithm are all branches of evolutionary algorithm. Many scholars have conducted research on these algorithms. Through continuous improvement, the performance of the algorithm has been improved, and the application field has been expanded. Therefore, it is necessary to discuss these algorithms. Characteristics, according to the adaptability of different application fields and algorithms, it will be very meaningful work to recommend different algorithms for use. After comparing these three algorithms, it is found that the DE algorithm has the best performance, and the algorithm is relatively stable, and repeated operations can converge to the same solution; the PSO algorithm has the second highest convergence speed, but the algorithm is unstable, and the final convergence result is easily affected by the parameter size. And the initial population; the GA algorithm has a relatively slow convergence speed, but in terms of dealing with noise problems, GA can solve the noise problem very well, but it is difficult for the DE algorithm to deal with this noise problem.

GA simulates the genetic evolutionary mechanism of human beings and organisms, which is mainly based on Darwin’s theory of biological evolution: natural selection and survival of the fittest. The specific implementation process is as follows: ①The individuals that adapt to the environment and perform well are selected from the initial generation population. ②Using genetic operators, the screened individuals are combined, crossed and mutated, and the second-generation population is generated. ③Individuals with good environmental adaptability are selected from the second-generation population and combined, crossed and mutated to form the third-generation population. The evolution continues in this way until the final population is generated, that is, the approximate optimal solution of the problem (Kumar et al., 2010). The principle of GA applied to this problem can be summarised as follows: 1) Firstly, a population *S* is determined. The number of individuals in this population is *L*, and each individual corresponds to *M* SNPs. 2) Set the fitness as *Fi*, and the fitness of each individual is:

$$Fi=\frac{Ui}{N}$$

*Ui* represents the number of samples that the population can uniquely identify, and *N* represents the total number of samples. Let the probability be *Pi*, and the *P* value of each individual is:

$$Pi=\frac{Fi}{\sum Fi}$$

Order the *P* values and calculate sort (*P*). 3) Individuals with high *P* value are selected and hybridised to generate new individuals. 4) The generated sub-individuals are placed into the original population, and the individual fitness and probability are recalculated through step 2. The corresponding number of individuals with low probability are eliminated. 5) Steps 2, 3 and 4 are repeated until the set number of iterations of the population is reached. 6) When the iterations are completed, the individual with the highest probability in the population is selected, which is the best SNP combination to be found.

The program written by GA can find the SNP combination that can distinguish all samples by computer language calculation. It has the advantages of high efficiency, convenience and high reliability, and it is a highlight of this study.

### Application Prospect of SNP Fingerprint Database Based on GA

In this paper, based on the SNP data of 533 *Oryza sativa*, 284 *Solanum tuberosum* and 247 *Sus scrofa* accessions, 100 core SNPs for fingerprint construction were selected from the original SNP data by GA calculation. All varieties of each species could be distinguished and identified by using these core SNPs. Each variety had its own fingerprint code, by which different varieties could be classified and identified. The two-dimensional code of *Manihot esculenta* Crantz fingerprint obtained by this method has been applied to field planting. Therefore, this method can be used for various species, has wide application and high practicability. In addition, the fingerprints obtained by GA were tested by the population structure. A small number of core SNPs used for fingerprint construction could basically restore the population structure of the original SNP data. Better restoration results were obtained in the populations of model crop *Oryza sativa*, autotetraploid *Solanum tuberosum* and mammalian *Sus scrofa*. For example, original SNPs from 533 *Oryza sativa* were to divide the population into nine subgroups, while the subgroup classifications of core SNPs were to divide the population into nine subgroups; the population structures were also highly consistent. Through the program written by GA, the core SNPs calculated had high accuracy; this set of core SNPs combination may provide new ideas and methods for identification analysis and genetic diversity analysis of each species (Semagn et al., 2014)^,38]^.

## Conclusion

In this paper, using SNP data of three representative species and the concept of GA, the SNPs with a high deletion rate and high single variation rate were finally screened out. A total of 100 SNPs that could distinguish all varieties in the population for constructing the model crop *Oryza sativa*, olyploid crop *Solanum tuberosum* and mammal *Sus scrofa* were obtained to construct the fingerprint. The proposed method is innovative and widely applied; it provides a new idea for genetic research and animal and plant breeding and has good application prospect.

## Data Availability

The datasets presented in this study can be found in online repositories. The names of the repository/repositories and accession number(s) can be found below: NCBI BioProject PRJNA171289; CNCB-NGDC BioProject PRJCA005945; GenBank GCA_000003025.6.
