# Supplementary material for: Feature Compression Applications of Genetic Algorithm
Source: Front Genet. 2022 Mar 8;13:757524. doi: 10.3389/fgene.2022.757524 (PMC8957834; doi:10.3389/fgene.2022.757524)
Supplement: Supplementary file 1 [file DataSheet1.PDF]

Construction of SNP Fingerprint based on a Genetic Algorithm

Meiling Zou[1,2,*], Sirong Jiang[2*], Fang Wang[3], Long Zhao[2,3], Chenji Zhang[2], Yuting Bao[2], Yonghao Chen[2], Zhiqiang Xia[1,2#]

Fig. S1 Method flow
Table S1. Names and sources of 284 Solanum tuberosum varieties
Table S2. 100 core SNP information of Solanum tuberosum
Table S3. 100 core SNP information of Oryza sativa
Table S4. 100 core SNP information of Sus scrofa
Table S5. Fingerprint coding of 284 varieties of Solanum tuberosum
Table S6. Fingerprint coding of 284 varieties of Oryza sativa
Table S7. Fingerprint coding of 284 varieties of Sus scrofa
Table S8. Two-dimensional code of Manihot esculenta Crantz fingerprint

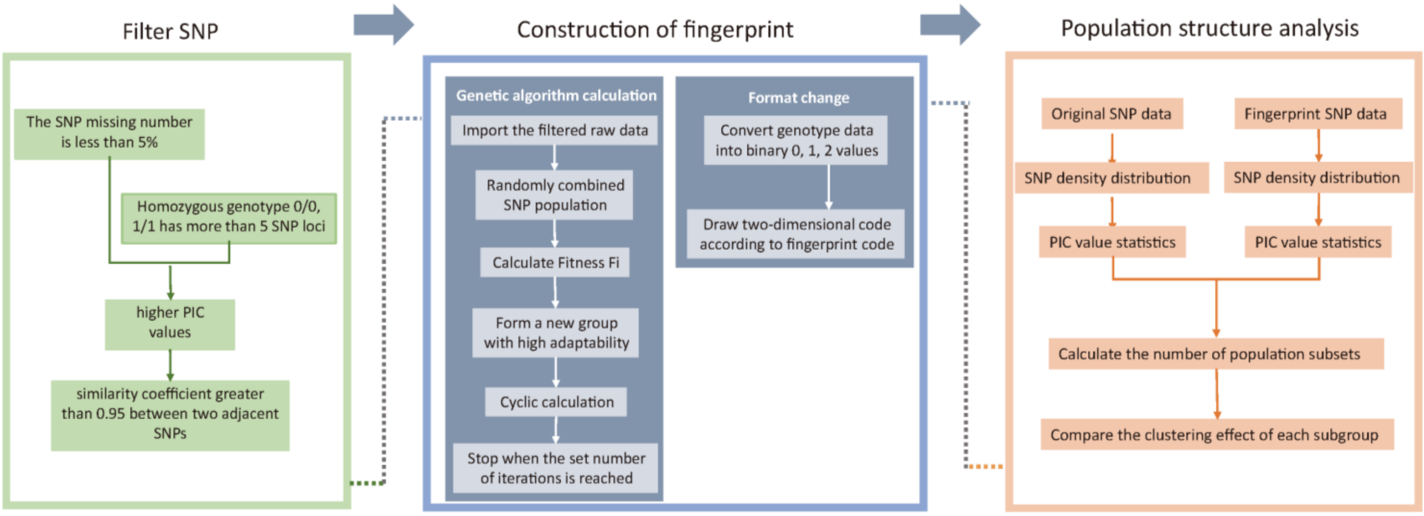

Fig. S1 Method flow

Supplementary table1. Names and sources of 284 *Solanum tuberosum* varieties

number	name	source	number	name	source
1	E60	CIP	143	CIP10-2	CIP
2	E62	CIP	144	RUSSIA1	Russia
3	E65	CIP	145	RUSSIA2	Russia
4	E66	CIP	146	Peru08-1	Russia
5	E73	CIP	147	Peru08-2	Russia
6	E76	CIP	148	KW-S-1	CIP
7	E80	CIP	149	KW-11	CIP
8	S25	CIP	150	KW16	CIP
9	E93	CIP	151	KW-22	CIP
10	E104	CIP	152	KW-40	CIP
11	E107	CIP	153	KW-41	CIP
12	R6	CIP	154	KW-45	CIP
13	S115	CIP	155	KW-59	CIP
14	R9	CIP	156	Dd-Ⅲ-2	CIP
15	R10	CIP	157	Cy-Ⅰ-5	CIP
16	R13	CIP	158	Cy-Ⅰ-6	CIP
17	R14	CIP	159	Cy-Ⅰ-16	CIP
18	Atlantic	U.S.A	160	Cy-Ⅰ-30	CIP
19	FAVORITA	Netherlands	161	Cy-Ⅰ-19	CIP
20	B1	CIP	162	Cy-Ⅱ-2	CIP
21	B3	CIP	163	Cy-Ⅱ-7	CIP
22	B4	CIP	164	Cy-Ⅱ-9	CIP
23	B5	CIP	165	Dd-Ⅱ-17	CIP
24	B6	CIP	166	Cy-Ⅱ-19	CIP
25	B7	CIP	167	Cy-Ⅲ-2	CIP
26	B9	CIP	168	Cy-Ⅲ-16	CIP
27	B10	CIP	169	Cy-Ⅲ-18	CIP
28	B11	CIP	170	Cy-Ⅲ-3	CIP
29	B13	CIP	171	Cy-Ⅲ-5	CIP
30	B14	CIP	172	Weishu 1	China
31	B16	CIP	173	taihong	China
32	B18	CIP	174	Calwhite	China
33	B24	CIP	175	Bojiao 2	China
34	B27	CIP	176	Shenyanwo	China
35	P5	Australia	177	BRDA	China
36	2340	China	178	Denali	China
37	NE303	China	179	FL1625	China
38	IP84008.4	CIP	180	Beifanghong	China
39	AK(CFK69.7)	Unknown	181	Hernes	China

number	name	source	number	name	source
40	DR-4	China	182	Yan005-432	China
41	T5	CIP	183	DT033	CIP
42	DR-6	China	184	J10622	China
43	DR-9	China	185	Canada-7	Canada
44	T6	CIP	186	kenebec	Unknown
45	W	CIP	187	Israel2	Israel
46	Zhuangshu 3	China	188	11	Unknown
47	A1	CIP	189	Pepo416-1	China
48	S1	CIP	190	RH	China
49	Zihuabai	China	191	BE13-3	Russia
50	Zhongda 1	China	192	BH2-5	China
51	A2	CIP	193	Ke9912-3	China
52	Qing05-12-6	China	194	Ningshu 1	China
53	06-26-7	China	195	Katadin×Ke 2000393-3	China
54	393382.44	CIP	196	Kexin 6	China
55	397073.16	CIP	197	Hu 9052-2	China
56	397073.16-1	CIP	198	Zhongshu 21	China
57	IVP101	China	199	Datongliwaihuang	China
58	S.st	CIP	200	NTB	China
59	A6	CIP	201	Tian0527—4	China
60	UK1	Britain	202	BH3-1	China
61	UK2	Britain	203	LK99	Unknown
62	UK3	Britain	204	Bf1-2	CIP
63	UK7	Britain	205	Bf1-1	CIP
64	UK12	Britain	206	Ningshu 1-1	China
65	A11	CIP	207	Qingzangxuan 2	China
66	Kexin 4×Aula	China	208	5P2-6	China
67	warba	U.S.A	209	5p1-1	China
68	A12	CIP	210	Ke3	China
69	Zhongshu No.4	China	211	E12	CIP
70	Ne 16	China	212	Zhengshu 8	China

number	name	source	number	name	source
71	D2	CIP	213	R3R4	CIP
72	D4	CIP	214	D8	CIP
73	D7	CIP	215	Zhongshu No.4-1	China
74	D11	CIP	216	D568	CIP
75	D13	CIP	217	Kexin 6	China
76	S5	CIP	218	Hu 9052-2-1	China
77	D14	CIP	219	Bf1-3	CIP
78	D16	CIP	220	BE13-8	Belarus
79	D20	CIP	221	H24	Unknown
80	W3	CIP	222	Kangyibai	China
81	W15	CIP	223	Kexin 5	China
82	W20	CIP	224	S11	CIP
83	W38	CIP	225	E23	CIP
84	F1	CIP	226	Bf1-4	CIP
85	S16	CIP	227	BH1-1	China
86	F13	CIP	228	Qingshu 10	China
87	F18	CIP	229	D600	CIP
88	E5	CIP	230	4p1-3	China
89	E8	CIP	231	Huayun	China
90	E11	CIP	232	5P1-5	China
91	E14	CIP	233	R1R2	CIP
92	E22	CIP	234	Herma	China
93	E32	CIP	235	L9810-18	Unknown
94	E41	CIP	236	0422-19	Unknown
95	E42	CIP	237	Canada-white	Canada
96	E43	CIP	238	Zangxuan	China
97	E52	CIP	239	PHUO35	China
98	Zhuangshu 1	China	240	D511	CIP
99	Superior	Unknown	241	NS78-1	China
100	Kaituozhe	China	242	Minshu 2	China
101	Pepo416	China	243	Canada 7	Canada
102	Shancheng	China	244	F8701	China
103	8089-115	China	245	Nunone	Unknown
104	Sebago	Unknown	246	L9901-132	Unknown
105	DT02	CIP	247	222	Unknown
106	283	China	248	Kexin 16	China
107	Jklway	Unknown	249	124	Unknown
108	Sebago-1	Unknown	250	63	Unknown
109	Youxiao	China	251	Kexin 13	China

number	name	source	number	name	source
110	Zaodabai	China	252	Yunshu 501	China
111	426	China	253	Qingshu 168	China
112	Bai 5-2	China	254	Zhengshu 8	China
113	Bintje	China	255	Qitaibaiyu	China
114	GARGE	Unknown	256	Zhongshu 18	China
115	Feixing	China	257	BE13-11	Belarus
116	LT-7	China	258	BE13-10	Belarus
117	UP-3	China	259	71	Unknown
118	Zhong A92-5-84	China	260	Zhongguo hong	China
119	JD202461-199	China	261	R1R3	CIP
120	Israel1	Israel	262	348	Unknown
121	Hua 525	China	263	Toylrum	Unknown
122	Chenggong	China	264	Canada 7-1	Canada
123	Chunshu 1	China	265	Ninglu 1	China
124	LT-5	China	266	Zhongshu 9	China
125	monona	China	267	26	Unknown
126	CIP09-1	CIP	268	4p2-2	China
127	CIP09-3	CIP	269	Qingshu 5	China
128	CIP09-5	CIP	270	Qingshu 9	China
129	CIP09-7	CIP	271	LC-98	Unknown
130	CIP09-10	CIP	272	Canada 3	Canada
131	CIP09-13	CIP	273	Kexin 8	China
132	CIP09-15	CIP	274	e5-1	China
133	CIP09-16	CIP	275	e5-2	China
134	CIP09-18	CIP	276	Ziyun	China
135	CIP09-20	CIP	277	288	Unknown
136	CIP09-22	CIP	278	Fuke 212	China
137	CIP09-23	CIP	279	Qingshu 8	China
138	CIP09-24	CIP	280	Ke200373	China
139	CIP09-27	CIP	281	43831	Unknown
140	CIP09-28	CIP	282	DY4-5	CIP
141	CIP09-29	CIP	283	Cy-Ⅲ-19	CIP
142	CIP10-1	CIP	284	ACD338	China

Supplementary table2. 100 core SNP information of *Solanum tuberosum*

Name	Chromosome	Position(bp)	Alleles type
chr1-1	chr1	1636796	G_A
chr1-2	chr1	1636802	A_G
chr1-3	chr1	2254231	T_A
chr1-4	chr1	4172595	A_C
chr1-5	chr1	4172603	T_C
chr1-6	chr1	7959076	A_G
chr1-7	chr1	8442560	C_T
chr1-8	chr1	8442633	A_G
chr1-9	chr1	9249040	T_A
chr1-10	chr1	9249062	G_A
chr1-11	chr1	9249071	A_G
chr1-12	chr1	10546764	A_G
chr1-13	chr1	15711076	C_T
chr1-14	chr1	19550524	G_A
chr1-15	chr1	19550535	G_T
chr1-16	chr1	20652161	A_G
chr1-17	chr1	20652222	A_G
chr1-18	chr1	23061138	A_G
chr1-19	chr1	25243255	A_G
chr1-20	chr1	32437310	A_G
chr1-21	chr1	32674744	C_T
chr1-22	chr1	32674782	C_T
chr1-23	chr1	32845237	C_T
chr1-24	chr1	37872002	T_A
chr1-25	chr1	37872046	T_A
chr1-26	chr1	46061071	T_C
chr1-27	chr1	54361477	A_T
chr1-28	chr1	54835631	G_A
chr1-29	chr1	54935815	C_T
chr1-30	chr1	56124886	A_G
chr1-31	chr1	59843576	A_C
chr1-32	chr1	59843578	G_A
chr1-33	chr1	72677285	A_G
chr1-34	chr1	72677545	T_C
chr1-35	chr1	75659577	A_G
chr1-36	chr1	82470124	G_A
chr2-37	chr2	22390169	G_T
chr2-38	chr2	41444009	T_G
chr2-39	chr2	44833860	C_T
chr3-40	chr3	7472728	A_T

Name	Chromosome	Position(bp)	Alleles type
chr3-41	chr3	8108664	T_C
chr3-42	chr3	13006192	A_C
chr3-43	chr3	15606418	G_A
chr3-44	chr3	24407952	A_C
chr3-45	chr3	61453178	C_T
chr4-46	chr4	11790795	T_C
chr4-47	chr4	12222513	G_A
chr4-48	chr4	12222543	T_C
chr4-49	chr4	14340231	G_C
chr4-50	chr4	25012979	A_G
chr4-51	chr4	28898294	G_T
chr4-52	chr4	40956127	T_C
chr4-53	chr4	49011568	C_T
chr4-54	chr4	49011717	C_A
chr4-55	chr4	49604686	G_A
chr4-56	chr4	52390501	A_G
chr4-57	chr4	65545220	A_C
chr5-58	chr5	9829961	G_C
chr5-59	chr5	11966126	A_T
chr6-60	chr6	8396171	T_C
chr6-61	chr6	22452351	C_T
chr6-62	chr6	24720304	A_T
chr6-63	chr6	40543028	A_G
chr6-64	chr6	51956116	A_G
chr7-65	chr7	18266568	C_A
chr7-66	chr7	18266623	C_T
chr7-67	chr7	29594862	A_G
chr7-68	chr7	33614770	C_T
chr7-69	chr7	33614778	G_A
chr7-70	chr7	34532284	A_G
chr7-71	chr7	43306244	C_T
chr7-72	chr7	43306365	C_T
chr8-73	chr8	15477173	C_T
chr8-74	chr8	22518863	C_T
chr8-75	chr8	27891636	G_A
chr8-76	chr8	27891975	C_T
chr8-77	chr8	32722357	T_C
chr8-78	chr8	36075130	C_T
chr8-79	chr8	36580988	G_A
chr8-80	chr8	54171517	G_T
chr9-81	chr9	1898888	G_C
chr9-82	chr9	2085938	C_G

Name	Chromosome	Position(bp)	Alleles type
chr9-83	chr9	11165111	A_C
chr9-84	chr9	52208326	G_T
chr10-85	chr10	33090712	T_G
chr10-86	chr10	38155103	T_C
chr10-87	chr10	50883576	C_T
chr10-88	chr10	55752709	G_T
chr11-89	chr11	2021684	A_G
chr11-90	chr11	9466690	G_A
chr11-91	chr11	22193188	C_T
chr11-92	chr11	22293434	G_C
chr11-93	chr11	27850273	T_C
chr12-94	chr12	14254380	C_A
chr12-95	chr12	14836865	A_G
chr12-96	chr12	25584480	A_G
chr12-97	chr12	29078628	A_G
chr12-98	chr12	29078633	A_G
chr12-99	chr12	42421347	A_T
chr12-100	chr12	48332880	A_G

Supplementary table3. 100 core SNP information of *Oryza sativa*

Name	Chromosome	Position(bp)	Alleles type
chr1_1	chr1	202360	C_T
chr1_2	chr1	223716	T_C
chr1_3	chr1	224009	G_A
chr1_4	chr1	257894	C_T
chr1_5	chr1	320875	A_T
chr1_6	chr1	401945	A_T
chr1_7	chr1	412634	A_G
chr1_8	chr1	430087	T_A
chr1_9	chr1	446797	C_T
chr1_10	chr1	546606	T_A
chr1_11	chr1	657035	T_C
chr1_12	chr1	657369	A_C
chr1_13	chr1	841900	C_T
chr1_14	chr1	843339	C_G
chr1_15	chr1	853734	T_C
chr1_16	chr1	957064	C_G
chr1_17	chr1	959818	G_T

Name	Chromosome	Position(bp)	Alleles type
chr1_18	chr1	1503750	C_T
chr1_19	chr1	1689721	A_C
chr1_20	chr1	1693595	A_T
chr1_21	chr1	2787795	A_G
chr1_22	chr1	3197919	G_T
chr1_23	chr1	4116510	A_G
chr1_24	chr1	4896087	T_A
chr1_25	chr1	4911632	A_G
chr1_26	chr1	6685775	A_G
chr1_27	chr1	6713964	G_A
chr1_28	chr1	6718661	A_G
chr1_29	chr1	9884814	A_C
chr1_30	chr1	10289731	T_C
chr1_31	chr1	10316496	T_C
chr1_32	chr1	11310137	C_T
chr1_33	chr1	12807160	G_A
chr1_34	chr1	13145818	C_T
chr1_35	chr1	14252598	T_G
chr1_36	chr1	14919131	T_C
chr1_37	chr1	15435236	G_T
chr1_38	chr1	15851165	G_A
chr1_39	chr1	16420525	T_C
chr1_40	chr1	18283979	C_A
chr1_41	chr1	19011272	G_A
chr1_42	chr1	20438101	T_C
chr1_43	chr1	20933371	T_C
chr1_44	chr1	21905055	T_C
chr1_45	chr1	26888824	A_G
chr1_46	chr1	30377546	G_C
chr1_47	chr1	31038588	A_G
chr1_48	chr1	35906130	T_G
chr1_49	chr1	43145243	G_A
chr2_50	chr2	2950405	T_G
chr2_51	chr2	13509070	A_G
chr2_52	chr2	16655858	C_T
chr2_53	chr2	22455542	T_A
chr3_54	chr3	1132091	C_T
chr3_55	chr3	1580530	T_C
chr3_56	chr3	26603228	G_A
chr3_57	chr3	35753598	T_C
chr4_58	chr4	5311552	G_T
chr4_59	chr4	17568250	G_A

Name	Chromosome	Position(bp)	Alleles type
chr4_60	chr4	24034846	T_A
chr4_61	chr4	29032673	T_C
chr5_62	chr5	6882306	T_C
chr5_63	chr5	8495908	C_T
chr5_64	chr5	8774369	A_G
chr6_65	chr6	5173943	A_G
chr6_66	chr6	9356517	T_C
chr6_67	chr6	9571703	C_T
chr6_68	chr6	12827892	C_G
chr6_69	chr6	28502626	T_C
chr7_70	chr7	6897384	G_A
chr7_71	chr7	7396550	A_G
chr7_72	chr7	7398562	G_A
chr7_73	chr7	8153616	G_A
chr7_74	chr7	11639310	A_G
chr7_75	chr7	14720584	T_G
chr7_76	chr7	16048329	T_C
chr7_77	chr7	20228876	G_A
chr8_78	chr8	3018865	C_T
chr8_79	chr8	19585162	C_T
chr8_80	chr8	20435008	C_A
chr9_81	chr9	464246	T_C
chr9_82	chr9	10351161	G_A
chr9_83	chr9	21201584	G_A
chr9_84	chr9	22385743	G_A
chr9_85	chr9	22773907	T_C
chr10_86	chr10	2048947	T_C
chr10_87	chr10	20807160	C_T
chr10_88	chr10	22124823	C_T
chr11_89	chr11	6527438	G_C
chr11_90	chr11	10569571	G_A
chr11_91	chr11	11251377	C_T
chr11_92	chr11	22829722	C_A
chr11_93	chr11	22893294	T_C
chr11_94	chr11	23714561	G_A
chr12_95	chr12	1580518	C_T
chr12_96	chr12	2077898	T_C
chr12_97	chr12	17399867	A_G
chr12_98	chr12	19281343	T_G
chr12_99	chr12	22406896	T_A
chr12_100	chr12	25953956	G_A

Supplementary table4. 100 core SNP information of *Sus scrofa*

Name	Chromosome	Position(bp)	Alleles type
chr1_1	chr1	10074730	A_G
chr1_2	chr1	36041827	C_T
chr1_3	chr1	295992465	T_A
chr1_4	chr1	296754899	G_T
chr1_5	chr1	301450249	G_A
chr1_6	chr1	304732537	C_T
chr2_7	chr2	11343685	G_A
chr2_8	chr2	16763122	G_A
chr2_9	chr2	18878461	C_T
chr2_10	chr2	44101938	G_A
chr2_11	chr2	48754193	T_C
chr2_12	chr2	50421722	C_A
chr2_13	chr2	51359926	T_A
chr2_14	chr2	112149330	C_T
chr2_15	chr2	155699640	T_C
chr3_16	chr3	16952136	C_T
chr3_17	chr3	20777466	G_A
chr3_18	chr3	67000106	C_T
chr3_19	chr3	121793568	A_G
chr3_20	chr3	128263741	T_A
chr4_21	chr4	15294232	G_A
chr4_22	chr4	27031069	T_C
chr4_23	chr4	75101454	T_C
chr4_24	chr4	140371138	C_T
chr5_25	chr5	12300919	T_A
chr5_26	chr5	71291159	G_A
chr5_27	chr5	73646229	C_T
chr5_28	chr5	73758248	G_C
chr5_29	chr5	82922536	G_A
chr5_30	chr5	103780766	T_G
chr6_31	chr6	8368719	C_A
chr6_32	chr6	13683379	T_C
chr6_33	chr6	16960211	C_T
chr6_34	chr6	22055182	G_A
chr6_35	chr6	40849088	G_A
chr6_36	chr6	112771374	G_A
chr6_37	chr6	113633465	C_G
chr6_38	chr6	125333197	T_A
chr6_39	chr6	135745626	G_T
chr7_40	chr7	20258205	T_C

chr7_41	chr7	78529596	C_T
chr8_42	chr8	13190851	G_A
chr8_43	chr8	34910776	C_T
chr8_44	chr8	78584340	C_A
chr8_45	chr8	134000339	C_T
chr8_46	chr8	141627617	G_C
chr9_47	chr9	6550053	T_A
chr9_48	chr9	17949671	G_C
chr9_49	chr9	38270961	A_G
chr9_50	chr9	70247799	G_A
chr9_51	chr9	120066006	A_G
chr9_52	chr9	123198953	G_T
chr9_53	chr9	123461256	C_A
chr9_54	chr9	127548053	T_C
chr9_55	chr9	142290517	G_A
chr10_56	chr10	7408298	C_T
chr10_57	chr10	9445033	C_T
chr10_58	chr10	9721992	C_G
chr10_59	chr10	18417719	C_T
chr10_60	chr10	19067162	G_A
chr10_61	chr10	20926403	C_T
chr10_62	chr10	22020295	C_T
chr10_63	chr10	36021860	G_A
chr10_64	chr10	39082659	C_T
chr10_65	chr10	41412411	C_T
chr10_66	chr10	42012167	G_A
chr10_67	chr10	44716984	A_C
chr10_68	chr10	46417202	C_T
chr10_69	chr10	47705206	C_T
chr10_70	chr10	49656347	T_C
chr10_71	chr10	50802622	G_A
chr10_72	chr10	52654253	A_G
chr10_73	chr10	54156362	A_T
chr10_74	chr10	54393372	G_A
chr10_75	chr10	54592021	C_T
chr10_76	chr10	58120920	A_G
chr10_77	chr10	67685349	A_T
chr11_78	chr11	80965441	C_T
chr12_79	chr12	38796400	G_A
chr13_80	chr13	799619	A_C
chr13_81	chr13	7961123	T_A
chr13_82	chr13	25197852	C_A
chr13_83	chr13	30196792	C_T
chr13_84	chr13	61953672	T_C

chr13_85	chr13	89322952	A_G
chr13_86	chr13	146959459	G_A
chr13_87	chr13	148509002	T_A
chr14_88	chr14	8592887	A_G
chr14_89	chr14	23119931	A_G
chr15_90	chr15	2651330	C_G
chr15_91	chr15	2976882	A_G
chr15_92	chr15	17028007	C_T
chr15_93	chr15	26704001	A_T
chr15_94	chr15	30248391	T_A
chr15_95	chr15	43078359	C_T
chr15_96	chr15	99036181	C_T
chr16_97	chr16	73148761	G_A
chr16_98	chr16	78521529	T_A
chr17_99	chr17	45060374	A_T
chrMT_100	chr26	16220	T_C

Supplementary table5. Fingerprint coding of 284 varieties of *Solanum tuberosum*

Name	Fingerprint Encoding
E60	00000222111101111001000001012222000012001101210010212220<br>0120000100022001000001010020220000102012000 0
E62	00022200111101111221220001020101020221001121210022210020<br>1000200200200000202000100001210000010202000 00
E65	22000000111101111012000221010201220010001102020001210022<br>2021222000000002202202112212120000100001000 0
E66	00200100111201111202002221200020020011211201010020122202<br>0200002122022001000020012210120200100210220 2
E73	00200000000102211201000221212222022002020101020012110020<br>0000022222222221010000222020101000200200000 0
E76	00111022222122200001220001012222020012000102020022110000<br>0200220222000001022220012012101000102000000 2
E80	00011011000101111001002111010021000012101001120020111100<br>0020200122011001020002002012100000202000220 0
S25	00000022111101111001000221200022000012011221010000110002<br>0022202100000001220002012212220000100110220 0
E93	22022100000121111202000002012001020022001102102200212201<br>2000000200011002000000122000100001120020000 2
E104	00000000111202211100002000000000101001102110211111021110<br>2020000010000000100000201001020202010000000 00
E107	00000000222211111100000000001000102021200210010112012001<br>2020000001100000100000001201020200010000222 00

Name	Fingerprint Encoding
R6	0012200022210112200000000002102012020111211220100101100000<br>22020010002202100000221221212020010000020012
S115	0000000222220112220100000101000120002120210221001211000000<br>00002020002222100112002221210000020001000000
R9	0022200022220111101200200100012222122102110101222211222202<br>00002020002222110000002001002001010200000022
R10	0002202200010110000222022201002210021100100111001201000022<br>00000011101122120000001210110000012022000000
R13	0020000011110112200200122222000122201100010102221012221020<br>20222000000020120000021001010200010000000000
R14	2221100022222112220100000101200100222120110102002211222200<br>10022210020000020220201002220020000020022020
Atlantic	1102202200010110000100222121000100002122110222110221000220<br>21000110002202102001201201110220120020000020
FAVORITA	0020002200020112201200222102000102221222220102222001222220<br>20020112200022120000011002010000222200200020
B1	0000002211120222220100000101200100202120110102000111222200<br>10012022220002200220201221200220220022000020
B3	0020020022220111100100020202200102202222212121001011000020<br>20000022202202120221021201010000210212000000
B4	0000000002221222220010020012100012020112011110200011100000<br>00200212200000120000001001200020010000100200
B5	1120000011110111100000200011010122221110112101002221000200<br>01000020002220100110201221212020220000000200
B6	0001102211110111100100000010002210001100122101000211000000<br>02020110000020100000202201212000022200000200
B7	2200020011110111100100000121012220202100112102001222002000<br>00001012202200200220002001110002020000000200
B9	1101100011112221102100011120000120201220010101002121220000<br>00200212202200100220001002000022010200002000
B10	0012200011110221100122211102002220200100121201001220220000<br>02200212202200102220002202220000010200022000
B11	2222202200010112222200022021022200202020210001221012000020<br>20000210000020100220002202010020022000000220
B13	0000000011110111100100000101000102001120112121222211000000<br>02020022202202102000000001011222210222022020
B14	0000020011110221100122200101000100001100112101001011002010<br>00000010000000100000001001020000020000000000
B16	0000000011120220000100200102121000001120110101001011000020<br>00022012220001102002221001020220010002022020
B18	0020000011110112222100200101000101202222220101001221000000<br>02002010002210122000011002010020020020222000

Name	Fingerprint Encoding
B24	000000001111011110010000020122010000120011012011102100022<br>00000010000000220000021201212002222220000000
B27	220222020001022000020001112102220200010011020122102200020<br>01002210000012121000222002022221002122000002
P5	220000001110011220012202220102220220110011010100211100002<br>20102212212220200220002201210201000200002200
2340	110000001111011110020000012120010200210211012100101122002<br>10000010010000122000001201010022020200002200
NE303	000000001111022110220020022100012002120011212100121000100<br>00120020000012102002221021020202010000200002
IP84008.4	220000002222211110210000010220012000110011010100021122100<br>00222210000020220000000202101100021002021102
AK(CFK69.7)	002000111111011220010022220000010000112212010100101100020<br>22202020000002200222001001210202010000100000
DR-4	002220001111011220012202220120220220112222210100001200000<br>00202010012200200220002011212000220200000001
T5	000220002221011220010000010100012002110022020100121200020<br>00022010000000110002021001010000000202000020
DR-6	220000001111011110010000010100010000210011210100101100000<br>00000010002200100002001001020000010000002200
DR-9	000000001111011220010002210100200220100011120122001200220<br>00200010000000200222001002210000010010000001
T6	000000001111000222020002222202220000112011010122102100020<br>20020020000000010220022201020000000202000000
W	000000001111211110010000010120012000210012010100122100022<br>11200000000012200210001021210020211220000000
Zhuangshu 3	002220001112022110210020010101220022212011020100221100202<br>10010010000000220000021022012012010200000002
A1	222000001112211002012200010100010000110221000100120122102<br>22000010020020202111001210210100002000200000
S1	000022112222022110220000020000220022111011010200101122001<br>20002222200020100110001221010000010122011102
Zihuabai	002002221112211221010020020100011200112211010100101100020<br>22220220002202200220002222112002112220022202
Zhongda 1	002222000001011110110000011002012020112021020100121022202<br>00002010002222120000022001012000010100012200
A2	001222001112111002012220020120010220101011100100121200101<br>22000222200000221220001001210201220202200022
Qing05-12-6	220220111111221111122200000120212010001110110101002101222220<br>00201012200000002022000200202002021022210002
06-26-7	000220112221022220010000012100220200120012010100102122220<br>21201202220220012000200102201020011000000022

Name	Fingerprint Encoding
393382.44	2200000011122111102111100102220100021100110201001211002000000201220000012200200100102222021000000020
397073.16	0000020011122111100100222101212222121100110121222011002002000001220000020000020112201020001000000001
397073.16-1	2200020011110111100200000102000122112100122222111002222022000101220110210000122121102000201200200002
IVP101	0001100011120111200211200100020100001202010102222022220020000102002000000000000001001210000020000020000
S.st	0002200011110111102100200101020101221201102122001211000200100001000000000000220001000110022112022100000
A6	00220002222221100201220002210001001021202101210020122200222222210012222200222201222210220012200000000
UK1	2200000011110112102200000221000102201212212202001001220002020000000220110000201100001000201010011110
UK2	2200020011110111120100022201200102001102010201001101222000000021200000002020000201001220200222000211222
UK3	0000000011120111120200222202000100021102210101002022200000002000220000020200200200021000002222010020
UK7	0000000021110111100100211101002200001000220101000011002000022020000000211010002100020100020111220220 0
UK12	0002200011120221100100022101002200021102110201220011002202002021001000020022002202201200202022220000
A11	2202200011120111100100200102020100021200110120221202002020000122001000020200222122121010021220220000
Kexin 4×Aula	0002200011112211102111000201220100001122110202002011000102201001000000212000020102101000001000200022
warba	0020000000012111220222000220200102021101120200000221220220102021002000012011000202201202022200002200
A12	0001100022200221120100122101200100022222112001221111220220200001000000001200000010010112020200212110 0
Zhongshu No.4	2222200022222111100100022101020100001101110202221112000200022012220002220022020222201000101202101102
Ne 16	0021100011112221102122022100000100111100210101002211000002200000000000010200102100112210001000000000
D2	0001102211110001120000122021000101221202210101002202222001200021000000212011100122000020022002010001
D4	2202220011111112200111100101222211001102222100002011000002022222000000000002202010110120001202002220 2
D7	2222222200022112222010002210122220220112011212000201000022220200220200202221200112222221000220222022 02
D11	1101202211111200220020022222221222101120012212100112122020002202122222202010002212220100200200222220 2

Name	Fingerprint Encoding
D13	000002111111011220010002220200220200110012012100201222000<br>2002002000000010222220202221110201002001102
S5	220000222211022110210000022102010200110211012100101100002<br>0020002000001010000000100101020021020000000
D14	220122221111011220010011110101010202110021012122101100000<br>2002201220000010000000122121002121002010002
D16	000200001111011110010001120100100001210021210102201200000<br>0222202200220012000000000201212221200000002
D20	000220001111011110000002210200220010110022020111222100012<br>1220201000110010200200200201000001002000002
W3	221000001112011220020002220002221002110011010100121022002<br>2202202000000220200020100101002001020020002
W15	000000222220022000010000020100100021110012011100100100000<br>2000222220002010222121220101220101002000002
W20	000220001111011000010022210202220200212011010111101100000<br>2202002220000010100022102001122011002020000
W38	000002001111011002020010020120220020120112022100122100200<br>0020222000002010000200000212020221002002211
F1	220000112121011112010020021100010220120021012100201100000<br>1220002220220012011000100102020020002000002
S16	000002001111211112020002220222100201222121020100121100200<br>0010002002000000200002200202000011022000021
F13	220220001111011110010001110102010200110021202122022122200<br>2020001002000112000202000102002001020202202
F18	000112221111211110010001110100010222010211212200101100020<br>0200212000000220200002100121102220202000000
E5	002220221111011110020000012120010020122021220100221100200<br>0000201000220010222020022122010001200002202
E8	002002000002222110210001110120220000212012210200121111002<br>0002002220112012000000100121002101000201102
E11	000220001112022111022200020222011102020021022122201122002<br>0022001220110020100020100022212001000202202
E14	222000000001211110000020022100011100111202011100200122020<br>2000102001222222222200100221022011000022202
E22	000111220001011112022200010121010000102211010111102100000<br>2002001220222112200220111201200021002000000
E32	222201001112022112010000020222012002120222012222102100000<br>2000001222110020000220100101020201000200000
E41	002222001111011110020000012101220200100111010202201122200<br>0000002000220212022100122022000201000020000
E42	000000221111211110110002210100100001110022010200201100000<br>0002001000000010000020200001020021102000002

Name	Fingerprint Encoding
E43	000000000001022112002222212120220220212200210000100100200<br>00000010000022221000202201222200210220000002
E52	222000000001011112020002212122220220020211212200200200200<br>00000022200000101000000201220102020022000000
Zhuangshu 1	020000221111022110012200010222010002110011020122101100022<br>00000210000010100000021011020000110000000010
Superior	000000002222011110010020012102010002110021010200102111002<br>00200212220000100002001201110002220000002120
Kaituozhe	000000111111011220010020010100012002210011010100101122020<br>00000110000010100220001001010002020000000000
Pepo416	000002001112011112010000010102010002110012010100202100000<br>00000210022220100002001111110000010000002200
Shancheng	000000221112011210010000000102010000110011210100201122022<br>01000010000000120000001101012002010000000000
8089-115	220000112222011222212200010202010000111211210100102100000<br>00000210002200120000001021010022110000200000
Sebago	002000001111111110012200010110012202110211020122222200000<br>00000210000000122000211021010010110200002200
DT02	110002222211011220021100022100220000010011012100101100210<br>00000220000000120000000001012000010200000020
283	000000221111011110010020010100010000110011010120002100000<br>00200010000002100000021011011000020000002200
Jklway	000000001111011220012200010100010000110212010100102100000<br>00002010020000100000021001020000010000000020
Sebago-1	000220001111011220010010010100012000220011020200101200020<br>01000010000000100000021001010000020020000000
Youxiao	000000111111011110010000010100012020210011010100101200222<br>00000120000020200000101011210000010020000000
Zaodabai	000001222220211220012202222100100000112011020122101100002<br>00000010000000100000201021020002010200000000
426	000000002222022110010000010200010200110211210200101100000<br>00000010000010100000000100101020201000020010
Bai 5-2	2202200011121111110010020020102010000112002210122101200000<br>00000210000020100220001001110200010000000000
Bintje	000001222222111112012220010122012000212011010200122100000<br>02022210002220100220001001210020021020022220
GARGE	222000002112012110010000012200010000210011010100202100012<br>01000010002200100000001001010020010000000010
Feixing	000222001112022110012220010100012000110011010100102200000<br>00100010000000100002021001010000010010000000
LT-7	001002002220011112020020011202010022212001210100101000010<br>22002010000020100000001111012002020000022200

Name	Fingerprint Encoding
UP-3	00000022222101111001110001012001201012001101010010110000<br>0000021000221012000000111121002221002000000
Zhong A92-5-84	00022000111200011021220001011001000211002101020010102220<br>02212221002000011000010122001202001220002200
JD202461-199	22200022111201122201000222010001200001021101201110120000<br>00200221000222010011202102101000202000000020
Israel1	22000000111101111001000001010001020001022120011112110000<br>00000012000220010022200222111100001002200000
Hua 525	00000222222102222021000001210001000012021101010010111102<br>00000001000222010000000100122002201000220020
Chenggong	00000011111102211001001002020201200012001021020010210000<br>00100011000002210022200100101200211002000020
Chunshu 1	00000111111101111022220001010022000011002102012210210000<br>20000001002000010000002100102000201000000020
LT-5	00022000222121122001000001022001000011001201010210110020<br>00000002001000010022020101101020001000022200
monona	22022222222211111100122122101012222002122212121112221000<br>022200211000000210011200202202222221222002200
CIP09-1	00000022111101111021000001220222002011002121010010122220<br>00000211002000211000020100101001001200200020
CIP09-3	00000022111101111002000001010022001011021100020010110020<br>00001001000220010000000201101020002010020210
CIP09-5	22222100111221111122000001210201000212001102020010110000<br>00221021002002011000200202220200000100000000
CIP09-7	00000022111202211201002001010001000021022101210010110022<br>20000000111002002020000010010202000100020000
CIP09-10	00000000222101111001000001020001000012002101010010220020<br>00000001000000010000002100101000002000022200
CIP09-13	00000022111111111001000001020022001021001211020010110020<br>00000001000000011000200101102200001020000010
CIP09-15	00000200111001122201000001010001000011002102010020210020<br>00000001002000010000000100101000001000000001
CIP09-16	00000100111001111001001002010022000011001221010010220022<br>00000001000000010000000100122000201000001100
CIP09-18	00000100111102100001000001010001001222001120210010120020<br>00010001000020010011002100101002001000020000
CIP09-20	00000200222000011201222000010201000022001121012210110000<br>00000001000220020000000100121000000200000200
CIP09-22	22000200111021111101000002020001000000002100011110110000<br>00000001000000010000000200101010001000020000
CIP09-23	00000000111101111001000001010201000011021201020010210021<br>20010001000000010000000100100000000100000000

Name	Fingerprint Encoding
CIP09-24	00022000111102211001000001011001000011001121010010122221<br>20000001000000010022000100102000202001000000
CIP09-27	002220001111022112010002212000010220110211010200101100200<br>000000022022221200020010010100000100202000 0
CIP09-28	000000221111222110210000010100010001110011220100101100200<br>002000100000001000000210010100020100000000 0
CIP09-29	001000221111022110010000020120222000110021120200101200100<br>002000200000202000002210210202020100202000 0
CIP10-1	000002221112011110010020012102220000110011010222002100021<br>200000100000000100002101001010000010120000 00
CIP10-2	000110111111222110010000010100010000110021012122102100000<br>012000000200022020000212220100202200000000 0
RUSSIA1	220001222222211111200001001020201220021001102010010210002 0<br>00002210000020100222001001010002012000000 00
RUSSIA2	000000002221011220010020010102010000110011210100102100010<br>010000200022001000010210010100002000000002 0
Peru08-1	000000221111222110212200010100222000111012110100102200000<br>000000100200001000000010210102000110000020 0
Peru08-2	000020002222011110010000010200012000110022010111201100000<br>002000100000001000020010010102000100000000 0
KW-S-1	000002002221022110010020010100010002110011210100101100000<br>000000100002001000000010010202020100000220 0
KW-11	000000001111011112010000010100010002210022010122101200202<br>000000100000011000000012012200000110000000 00
KW16	220000000222201122001220002011001000012002111012210220020 2<br>00000010000000210000000100101020201000000000
KW-22	000000002222011112010020010102010000120211112100102100202<br>002100200000001000020012010100000120000002 0
KW-40	000221222122011112222200010110010000110010110100102100002<br>000202100022001000022210010201000100002001 0
KW-41	002002112221011110012202220120220000120011022222201200022<br>002022222000002000020020000111020200210002 0
KW-45	000002002222011110012200010220010000110012210100102100001<br>000000200000001000000210010100000100020220 0
KW-59	000000221112011110012202210110010000210011010100101200022<br>000000100000001000000210112102000100102000 0
Dd-Ⅲ-2	000002002222011001020020010100220000120002021120101211012<br>100000111000000102000021001020200000000200000
Cy-Ⅰ-5	000001001112011111010000000100010000100011011000121000001<br>000000000220001022200210000100000100000000 2
Cy-Ⅰ-6	002220001111011112210000011122010200112011212202102000000<br>200020100100001020000210210100020100220002 0

Name	Fingerprint Encoding
Cy-Ⅰ-16	000002001111011112212200010121010002100211012200102200002<br>2220202220020010000000100122020001200201100
Cy-Ⅰ-30	000001002222011111010000010200010000110011011122101100002<br>0000001222002010000000100101020201002000000
Cy-Ⅰ-19	000000001111011220011100010120010002120010211200102100200<br>0000000222000010100000110021002201000000000
Cy-Ⅱ-2	000000001111200220010000011121010000200020010122121200200<br>2020001222110010200000102102020102000020000
Cy-Ⅱ-7	222220001111011222210000010200012022110011010200101100000<br>0000021001000010200002100100002001000000002
Cy-Ⅱ-9	000000221110000112010020000100010110100011110100101100000<br>1022002000002020022000200101000021201012221
Dd-Ⅱ-17	000002001111011221020000010120010002020211212100102000002<br>0200001222000010200002100102220101000201100
Cy-Ⅱ-19	000000002221211111012200020110010000100011211200202000001<br>0020001222000010000202120101010001000200010
Cy-Ⅲ-2	000220002222012110022200010122222000120020212200201100200<br>0000200222000001000012200121222001000020000
Cy-Ⅲ-16	000000001111000111010010010201010020210211212000100100000<br>2200221222000010200000100102200221200200000
Cy-Ⅲ-18	000220001111211111110022210121010000100222012100101122001<br>0000000222220010000000100121120101200200000
Cy-Ⅲ-3	002001001111000112010000010120010000120012212122101011001<br>0000202002000011000000100122020101000202200
Cy-Ⅲ-5	000000002221022220110000012100012022110010012100102100000<br>0010201002222010000000200101002001000000000
Weishu 1	002000002222122222212200010220011120110211210222101100002<br>0200201002002020000002101202100000102020022
taihong	000002001112022112210000021100010000212011212100101200000<br>0000001002000020022200100111200001100000000
Calwhite	002000000001000000200000010202010020220212020122101122000<br>0200002002000012200001100202002002020010020
Bojiao 2	000001000001211110220000020122010002110021010100201100000<br>0000002112110020022000202220220202121000112 0
Shenyanwo	002001111111011002000002220100220000100011010200100100012<br>0221001222110000000200202112200102200200000
BRDA	000000001111111110220010020200012022110011010100101200000<br>0220012000001210022000100201000201000000020
Denali	000220221111022220210022220200220222110020220222101000002<br>2200201002222020122000200101220000200010020
FL1625	112002001111011220220020010202010020120210020200101111000<br>0222020100200022000000210110211000110000002 0

Name	Fingerprint Encoding
Beifanghong	0000020022210111100100200122000100021100212122002012000020200021000220010000002100102000002000000020
Hernes	0000000022210112200100000101000120201100112101221021000020020201002000020000200100101200222000000010
Yan005-432	0000010011110221120122022221002202201102211221001011220022221201001220110222200101201012201000210022
DT033	0000021111110222202100000201000100001200110101001011000020000002000000010000000102101000001000020002
J10622	0000000022212001122100022222220121201220112121001021220020020201000000210200020100201220001021101110
Canada-7	0020000002221222220120000000100012220110211020100101100200000220100200211000020010110000220100000222 0
kenebec	0000002222220112222122000101220121201102110101222012000220220001000222112000000100201222202201001120
Israel2	0000020011111111122200000202000121211102110102000211222000220001000220010200012200201222101102200020
11	0000022222210111122100200102200102001200111111221001000000000001002110020000022100101002001000000000
Pepo416-1	0020020011120111112100200101002200021100112201222022220000221200002002010000020101102002121000020002
RH	0020000011110221100122000102000100021102212202001012110100200001000220110000000002101110101020000220
BE13-3	1120000002221011110010000011102010000210012010100201100011220000100000001000000110010200202100120000 0
BH2-5	0020000002221211000020002211000012000110021210122211022000022000100000001200000220220202000100012002 0
Ke9912-3	0010000011122111102100000101122210001200120101221211000000120101000000012000000100011200221000002200
Ningshu 1	2200020022212111102100022101022221001100120101002221000000002001000002110200002201210000011200022220
Katadin×Ke2000393-3	2200000011120112200100100121020110022100120221001211000001100022000000010022000100111000002200002200
Kexin 6	1122202211122110022100000101200100221120111101222011000222202221002002220022000120122202211122221102
Hu 9052-2	2220022211110001122100000201020120022120012101001211222000220002002110012022200122121002221202002220
Zhongshu 21	0000000021220111100111000202010010110021010100101122110000001100000011020000012020120022100002220 0
Datongliwaihuang	2200020011110221120122000201200120201122110101221212001210200001220000010011000020221000002220000001
NTB	1112220022211111100111000222000120001100120101001011000200200001000000002000000210010102100120002220 0

Name	Fingerprint Encoding
Tian0527—4	0000000011110111100100000112000100011100110101001012112002000001000022010000002200102200021201000000
BH3-1	2000020022100111110100000002010100202200110222111212000000000000110000010200000100102010000202000000
LK99	1100000011121222200100000101100120001120110202001012220200000001002002010022000100121000201001002000
Bf1-2	22122022111112122220222000002002202001220220001222222000221202001222220000200020200102002201210100022
Bf1-1	0002002222210221100000000101000100002112212101001011000000200001000000220000001120101020001022002200
Ningshu 1-1	0000022211110112200100200011020101021120210001002012000202000011000002010022200002101002001000202100
Qingzangxuan 2	2200020011100111100100022202000120002100121102002011001100000021002000012000000122101200011022000020
5P2-6	1122220001111220022021220221010001120211211202010020112222021001010000002120000222222000000001120010020
5p1-1	2212200011120111102122000102220120021100112201001211002202020001000002112000000100101000001000002200
Ke3	22200022111112221100211000101022200101222110101001112000000202201220002012011220200202000121020222220
E12	2202000011110111120100200122000100001100110102001011002000000021000020010022002100102000101000020010
Zhengshu 8	0000000011110221100100000111000120021100110101001011002010020021000000012000000100100200001000020000
R3R4	0000021111110221100100211102002200001220110121221011000000000001000000110000000100101100101001000020
D8	0020012211111000000122000101202211001122210101000111000000002221002000020022000100101200222020000002
Zhongshu No.4-1	0002200011110112220111200122000100001100120101001211000000200002220000010000001102101022101002001100
D568	0000000011110111122122000101200102101120210121001221002222000202110002012000000122121220021002000002
Kexin 6	2200001111120111102122000101000122001220221101021011000200102012110022010022210120201002021102020200
Hu 9052-2-1	2220000011112111120100222101202200001200110101001221002000002020200220112200010102222002021000200020
Bf1-3	220002001112222211002000000020201000111200121020012110002212220020020000020200020100101001001200222212
BE13-8	220220221111121111001112111122002200001100112121001011220202021101220002220000021102121121001200020002
H24	2201100011110111121100000122020100201222200202111211000210002002220002211000000122121210202220022210

Name	Fingerprint Encoding
Kangyibai	110002001111211110011120010110102000210011010100101200020<br>0000222000001212022002100101000011012202200
Kexin 5	002002221111211111211222221020001002011201102010022110020<br>02022111002110212000020102201222002000012222
S11	002222221111011110010000010100222000110021120100101100222<br>0220101001222010022100102101000001002202200
E23	000220221111022110210001110100220000122012020200201100002<br>2020001000000222000020100021220022202002200
Bf1-4	112220002221011110010000012120220020110201010100121200200<br>0000021110000010200020120121000021001000000
BH1-1	220000002221022220012222212120010000210211201100201120000<br>0100002000000220200002110202000021100000000
Qingshu 10	220002221111222220010000002100010000110112210100101100200<br>2020011000221210000202200201000201000220000
D600	220000021111211110010002220100010020112211010100201222200<br>0000202000000012200220100112020001000002222
4p1-3	112202001111211110110020012100012000212011012100101022000<br>0220101000000012222100102221020001021100020
Huayun	110000221111011110010000010122012000110021010100101100222<br>0010001000002010200002100101001201200000000
5P1-5	000000001112211220020002210100102220102111012100121122220<br>0000201000002012222001200220202001200022200
R1R2	220000001111011110010000010120220021210001011100101122000<br>0201001000110010000020202201202021000020000
Herma	220002001111211222022200012200012000110221112100001100020<br>0200121000000222000000222020200202001100000 2
L9810-18	110000002222122110012201110100220100212012011200101111020<br>2200201002000010000002100111202022000000020
0422-19	000000002221211220010020022100010000110021010100100222210<br>2000121222000222000200122101020221220200022
Canada-white	002002221111222220010000020200012020120000020100102122220<br>2200202001000212000200101201000002000020022
Zangxuan	112220001112022222010012220100012100210021220200121100112<br>0000000002000012200002112121200021002200002
PHUO35	200000001110011122020000012002010000100012010222101111022<br>0000121000000000000000100101210201000002200
D511	002000001112011220010002220202011000100021010100101222000<br>0000022000000220100002110120122021000000000
NS78-1	000000002221011110012200022000220002212011010122101100010<br>0002001221000020000010200101002001200020001
Minshu 2	002002021111211220020002222102222101220011012111111122200<br>2220022220022210200222000120020001000022202

Name	Fingerprint Encoding
Canada 7	0022200011122111100100000202020122101100110102001211002202002021000222211000220102111000001020010000
F8701	2211100022212121100200000101020120021100110202002011000000200001000220010011000120101002001220020000
Nunone	2202202222210111100100000102021010002100112102001011000200210002000000012000002100101000221000200020
L9901-132	0000021111112112200100200222001000021122110102001011000002020022220110211000020200111200022020020000
222	2220000011112221122100000201000120002200121201002022000200022011002000211000101110120000001200220020
Kexin 16	0002200022212221102100100121000110221001122101001212000000012001000002012022202100121010002220000000
124	2222200011112112220100200121000100221110110201222210221000000002000002222022000221221100001000000001
63	2200002200012221100200000202210100021100110201002122222020000001220000020022000000201222001120202002
Kexin 13	00222022111221111100222100221000102011100210101001021000001022001221110220011202200201022001020102202
Yunshu 501	00000200222202211002220001020201120021000101011110200000020020210020000100222022001210002211100000000
Qingshu 168	1122222211110111122122022101000100021102010102002011222200002222000220022200022102200000121222020020
Zhengshu 8	2202200000010111102100122101002220021200010101001211002000200102000002212022000100221002222002000000
Qitaibaiyu	0000000011110121100100000101000100001100212101001011000010000001000000010000202100101000001001002200
Zhongshu 18	0000000022210111120122000201120102202200120101001011020000000201000000210000200100101000001000000000
BE13-11	0000000022210221101000022020022200202220210100221011111200222000111000011000000122021201002220200012
BE13-10	000000001111011110200000000000022000011001201012212210010022020022222200110000000102220201001200000022
71	0000000222210111120100000101000100001102110201001222000000022001000220210000012100210001101222020000
Zhongguohong	0000022211112002202222022101001002011100112100222012220002000221002000210212000220001202021020200002
R1R3	2200000000210111100100122101020100021102120122111011000202002001000220010022021202221200101002020001
348	0000001111112112200111000221002200001100110202001111000100020001002001010222010122101001001100110002
Toylrum	0000002211110221102100200221220122001100211102221221220110202201110002220200000100101202001002020000

Name	Fingerprint Encoding
Canada 7-1	00222002111121100022000002010001100211001120020010112220<br>00000202220002211000001122101001202020002202
Ninglu 1	00222000111101122020000002210022021001001102012200200002<br>22200001220002112022020212101000100220201112
Zhongshu 9	22200000000022220000100011101000102201101112221001101000<br>20221220100222011021110010220200020110220110
26	00022000122101100002000001020001020211001202010010110000<br>00000022100102011002202010010110001202000222
4p2-2	00000000111121122201000001210001220221021101210012112200<br>00020022000220010000001100111000201000200020
Qingshu 5	00011200222111111001222001222001020220102211010010110000<br>20201212222222021000000010010120102121000000
Qingshu 9	00000222111101122221000001020001000011211101212220210000<br>02020001000000212000020222222000202020010000
LC-98	00000022111121111021220001010001112011221101010010112202<br>01100001221002212200000200200000220101220000
Canada 3	00100200111101111022000002010022000011021100000000110001<br>00102121000000212200021200202012002000100000
Kexin 8	11000200111111111000112001010001000011020101010010110000<br>02200001220220020022000102101000201022200000
e5-1	00200011111101122001000000210110020011002111012210221102<br>22000002000000000020002012220100012110022002
e5-2	00022200121101122001000001020001200021001201010010110000<br>20000000100000000100000001001010200010200000
Ziyun	00022200111101111022000001022122220211202212010010110000<br>20020101200000012022000220121010001000200000
288	22200200000111111021000221010001001022022101010020120000<br>02200002001001212000201200021220001021000002
Fuke 212	00011000111002211022001002012022020011202101002210110002<br>02022020000000222000020010010120200101220000
Qingshu 8	22011022111221111001220002012022020211001121020012110001<br>00022221220220220000002210020000201102222000
Ke200373	00200000111101111001000001210002200111201102012220210010<br>20002001222000210100020001211000001000000022
43831	22122000000121111001220221000001000011200101022220100002<br>20002002000000010000020100201122201020022200
DY4-5	22200022111102211101220001010201100012220102020011010000<br>02020101002220000022100022021120001000020000
Cy-Ⅲ-19	00000200222101111101220001010001000011001101220010200000<br>10020000221220010000202100101020101200202100
ACD338	11000000222102222001002001010001020021002201000010122201<br>00000001000220010000002100201000201000000000

Supplementary table6. Fingerprint coding of 284 varieties of *Oryza sativa*

Name	Fingerprint Encoding
Wanlixian	00001000001100110000000010111000011100000001100000000000<br>1111100110000100001010000000100000000110000000
Feidongtangd<br>ao	11111111110010001011111000001111000011111001111111111111<br>0001011001111111111100011100111101111111111001
Hengxianliang<br>chun	00000000001100110000000010000000011100000001100010000000<br>0111100110000100000010010001000001011100000010
Leihuozhan	00000000001111000100000101110000111000000011000000100000<br>1011100110000100000000000001000000000110001000
Baikezaohe	00000000001100110100000101110000110100000011000010100000<br>0111100110000000000000100001000001011100000100
Haomake(K)	11111111110010001011011100001111000111111001111111111111<br>0000011001111111111100001110111101111110110001
Sanqishi	11111111110010001000011101111111100001111100111111111111<br>0000010001111111111100001100111101110001101 01
Niankenuo	11111111110010001011011100001111000011111001111111111111<br>0001011000001111101100011110111101110011110 01
Yangkenuo	11111111110010001011011000001111000011111001111111111111<br>0001011001111111111100111100111100110011110 01
Putaohuang	11111111110010001011111000001111000011111001111111111111<br>0000011000001111111100011110111100111111110 01
Guangluai4ha<br>o	00000000001100110100000101110000111000000011000000000000<br>0111100111000000000000110000100001000110000100
Xiangdao	00000000001100110100100101110000000110000001100000001010 00<br>0111010000000000000000101000010000100000000000
Younian	00000000001111000000001010000000000010000000110000010001 00<br>1110100001111000000001110000100101100110000110
Wukezhan	00000000001111000000000100000000010110000000000000100000<br>1100100100000000000001100000000000101000100110 0
Zhongnong4h<br>ao	00000000001100110100000101110000110100000011000000000000<br>1111100001000000000001010000100000000110001100
Minbeiwanxian	00000000000000100000100100000000011100000001100000110000 0<br>0100100100001000000110100000000000100011100010 1
Hongmisanda<br>n	11111111110010001011111100001111000011111001111111111111<br>0001011001001111101100111100111101101111111001
Yuyannuo	11111111110010001011111100001111000011111001111111111111<br>0000011001111111111100001110111101110001100 01
Maweinian	00000000001100110100000011110000000100000001100000000100 0<br>1111100001000000000010000001000000000000000110
Zinuo	00000000001111001111000101110000000100000001100000000100 0<br>0110101111000000000011000000000000000000000110

Name	Fingerprint Encoding
Beizinuo	000000000011001100000000101110000101000000110000000100000<br>01110010000000000000010100001001000000000001101
Dom Sufid	000000000000001011111110000000001100111110011111010101<br>0001011000110111111101011110111001110011110011001
xiangaizao10	000000000011001101000001011100001111000000110000000001000<br>011110011000000000000001000010000100011000010 0
Biwusheng	000000000011001101001001011100000010000001100000001000000<br>01011001111100000000000100000001000000000011000
Yizhixiang	000000000011001101000000100000001101000000000000001100000<br>1101100110000000000001000000000000101011000101 0
Xiaobaimi	000000000011001101001001000000000101000000110000000100000<br>0110100111000100000000110000100001000110001110
Zaoxian240	000000000011001101001001011100001110000000000000000000111<br>0111100110000000000000110001100001000110000101
Jing7623	000000000011001101000000100000001110000000110000000100000<br>0111100001000000000000110001100001000110000110
JinnanteB	000000000011110000000100101110000110100000000000000000000 0<br>1011101111000000000000100000100100000000000110
Funingzipi	1111111111001000101111110000111100011111100111111011111<br>000001100100111111110001111011110011110111001
zhenshan97B	000000000011001101000001011100001101000000110011010000000<br>0011100111000000000001000010100101000110000100
Qingsiai16B	111100000000010100000000010000000111100000000000000000000<br>0111100110000001100001000001000010100000001010
Weiguo	111111111100100010111111000001111000011111001111111111111<br>0001011001001111111110000110011110111111111001
Dianrui409B	000011111111001001000011000000000111100000110000010000000<br>101100011000000000000010000000010100000000000110
Liaojing287	111111111100100010111111000001111000011111001111111111111<br>0000011001001111111110001110011110111111111001
Huhui628	111111111111100110100011100000000000000111110011111111111111<br>0001011001001111111110011111011110111110111001
Gerdeh	111111111100100010111111000001111000011111001111111011111<br>0001011000111111111101111110111001111111111001
88B	000010000000001010000100010000000000010000001100000000000<br>1010000000000000000000111000110001100000000110
Xianghui91269	11111111110001000111100011110000000010000001100000000001000<br>0010101000000000000000101000010000000000000000110
Longhuamaohu	111111111100100010111111000001111000011111001111111011111<br>0000011001111111111110001110011110011001111001
Lucaihao	000000000011001101000001011100001101000000110000000000000<br>0111100110000000000100010000100001000110000100

Name	Fingerprint Encoding
Zhonglouyihao1	111111111100100010111110000011110000111110011111111111000001100111111111110011111011110111001111001
Yelicanghua	111111111100100010110010000011110000111110011111011111000001100100111111110001111011110111111111001
Shufeng101	000000000011110010001001000000000111000000011000000101000111110011100000000000010000010000110011000110 0
Chengduai3hao	000000000011001101000001011100001110000000110000000000000111110011000000000000010100001001010000001000110
Sankecui	000000000011001100001000111100001101000000110000000000001100110110000000000000011000000000100001000011 0
Gongju73	000000000011001101000001011100001110000000110000000000000111110000000000000000010100001000010001100001 10
Li-Jiang-Xin-Tuan-Hei-Gu	111110000010010001011011000001111000011111001111110111110000011000111111111100001110111101111110110001
Jiabala	000000000011001101000001011100001110000000110000000000000011100000000000000000010100001000010001100001 10
Taishannuo	000000000011001101000001011100001110000000110000000010001111100000011000000000101000010000100100100011 0
Guichao2hao	000000000000010100001000100000000111000000011000000000000001100100000000000000001000000100001010000000101 0
Huke3hao	000000000011001101000000100000000111100000001000000000000011111000000110000000000110000100101000110000100
Teqingxuanhui	000000000000010100001000011100001111000000000000000000000101001000100000110001100000000000100000000110 1
Huangsiguizhan	000000000000010101111000100000000111000000001000000000000001100011000000000000001000000100101000000000101 0
Xiangwanxian3hao	000000000011001101110000011100001101000000010000001000001011100000000000000000101100000000101000000100011 0
Taizhong65hao	000000000011001101000000111100000001000000011000000000000111110000100000000000101000000000100000000001 10
Zaoshunonghu6	111111111100100010111110000011110000111110011111101111100010110000011111011001111001111011111111100 1
Jinyou1hao	000000000011001101000001011100001111000000010000010010001011100110000000000000100100100001000000000111 0
AZUCENA	111111111100100010110110111111110000111110011111101111100010110011111111111000001101111011111111100 1
Chengnongshuijing	000000000000010100001000111100001111000000010000000101000111100011000000000000011010010000100000000010 10
PeiC122	111111111100100010001000100000000000011111001111101001000101010001001011111101000100110000000000111110
Guihuahuang	111111111100100010111110000011110000111110011111111111000001100000111111110001110011110111111111100 1

Name	Fingerprint Encoding
Momi	0000000000110011010010001111000011010000011000000101000<br>11010001100000000000001101001000010000100001010
Xiushui115	0000000000000100010000010000011110000111110011111111111<br>0001011001001111101100011100111101111111001001
Sanbaili	00000000001111001100000010000001000100000010000010001000<br>0101100101001000000000110000100001000001000100
Jindao1hao	1111111111001000101101100000111100001111100111111111111<br>000101100111111111110011110011110111000111001
Dandongludao	1111111111001000101100110000111100001111100111111111111<br>000001100011111111110000111011110111001110001
Liusha1hao	0000000000110011010000010111000011100000011000000000000<br>0011100000000000000101010000100001000110000110
Bawangbian1	00000000001111000000000001111000000010000011000000001000<br>0110100000000000000001010001100000100110000100
Shan-Huang Zhan-2	0000000000110011010000010111000011110000000000000000000<br>10101101100000000000010000000000101100000000001
Dongtingwanxian	00000000001100110000100101110000111000000110000000001000<br>0110100110000000000001100001001010001100000000
Yangdao2hao	11111111100001010000100010000000100100000001000000000000<br>1100000000110000000011000001000000000000000110
Zhengdao5hao	11111111111111100001110010111000011010000011000000001000<br>010010011000000000010110000100001010000000100
Jing87-304	0000000000000100010001010000011110000111110011111111111<br>000101100100111111110001110011110011000111001
Sugeng2hao	0000000000000100010001011000011110000111110011111111111<br>000001100000111110110001110011110111111111001
Hongwan1hao	0000000000110011010010001000000011110000011000000000000<br>11001000000000000001001000000000001000001001110
Liushizao	00000100001111001100000010000000100010000000000000000000<br>0001100110001000000011000001001010000000000000
Muxiqiu	00000000001111000000010110000111100001111100111111011111<br>000001100000111110110001110011110111111111001
Taizhongxianxuan2	00000000000001010000100010000000111100000001000000100000<br>11110001100000000000001000001000110000000011000
Dangyu5hao	00000000000001010000100011111101001100000001000000000000<br>11110000000000000000001000000000000000000000110
Swarna	00000000001100110100000101110000111000000000000000000000<br>10101101100000000000010000000000101100000000001
Youmangzaojing	1111111111001000101111100000111100001111100111111111111<br>000101100111111111110000110011110111111111001
Laohuzhong	00000000001111000000010010000111100001111100111111011111<br>000001100000111111110001110011110111111111001

Name	Fingerprint Encoding
Nantehao	000000000011110000000000011110001000100000001000001000000111010011011000000000001000010000100000100000
Esiniu	0000000000110011010000010111000111010000011000011000000101110010100010000000100000010000100011000010
cunsanli	111111111100100010110111000011110000111110011111101111100000110011111111111000011001111001100011001 1
Huangkezaonian	00000000001111000000101100001111000011111001111110111110000011001001111101100011100111101111111111001
Nantiangangjiu	00000000001111000000100101110000111000000110000101000001011100000000000000001100000100000000111000011 0
Benbanggu	00000000001111000000100101110000111100000110000100000001011100000000000000101100000100000000111000001 0
Qitoubaigu	00000000001111001100000101110000000100000001000000001000001110000000000000000110000000100100000000011 0
Muguanuo	11111111110010001011111100001111000011111001111111111100000110011111111111101011101111010111011000 1
MOROBEREKAN	1111111110000101000010001000000111100000000100000001000001011000111000000000001000001000000001000001000
Hanmadao	000000000011001101000000111100000001000000110000000000001111100001000000000001010000000010100000000001 10
Heidu4	00000000001111000000100101110000111000000110000100000001011100000000000000001100000100000000011000011 0
Gaoyangdiandao	1111111111001000101111100000111100001111100111111111110000011001111111111100001100111101111111111011
chikenuo	000000000011001100111110000011110000111110011111111111100000110001110111111000111101111000011111101 1
Haobuka	111111111100100010110111011111110001111100111111111111000001000111111111110010111011110111110110001
Shanjiugu	111111111100100010110111011111110000111110011111111111100000110001111111111001011101111011100011000 1
Fanhaopi	000000000000001011110101010000000001011111110000000001000011001011011100000000101100101101110000001000 0
Lixinjing	00000000000001000100010100000111100001111000111111011111000001100011111111110001110011110111111111101 1
Sadu-cho	00000000001111000100000001000000111010000011000011100000011110010000000000000110000010000100011000001 0
IR64	11111111110010001100011100001111000011111001111110110110000011000111111111101010110111101110011100 01
Dongtingwanxian	000000000011110001001000100000001101000000000000011011001000100100000000000000110000000001010110001100
Babaili	11111111110010001011111100001111000011111001111110111110000011001111111111100111100111101110001100 01

Name	Fingerprint Encoding
Qiyuexian	010000000111110010001001000000000110100000000000000100000<br>01001001100000011000110010000000101000000000110
Yuyannuo	111111111100100010111111000011110000111110011111111111<br>00000110011111111111100001110111101111100110001
Mamagu	000000000110011010000001111000000010000001100000000000<br>11111000010000000000101000000010100100000000110
Qingke	000000000000001011000001011100000001011111110000001001000<br>11101000011100000000100000000000001000000001000
Hongkezhenuo	111111111100100010110111000011110000111110011111111111<br>000001100001111111111011111011110111000110001
Mowanggunei	111111111100010000000000111100001101000000000000001000100<br>11001101100000000000011000001000000000000000000
Baigedao	000000000110011010010010111000000110000001100000001000000<br>01011001111100000010001000011010000000000011000
Tieganwu	000000000111100000010110000111100010001100111111011111<br>00010110000011111011000111001111011111111111001
CYPRESS	000000000000010100001000100000000111000000011000000000000<br>01110001100000000000010000001000010000000001100
Sanlicun	111111111000010100001000111100001111000000000000000001000<br>10000001100000000000011000011100010100100010000
Meihuanuo	000000000111100110000001111000011010000001100000000001000<br>11101001110000000001000100001000010000000000100
Lamujia	000000000111100110000001111000011010000001100000000001000<br>11101001110000000000010100001000010000000000100
Magunuo	111111111100100010110111000011110000111110011111011111<br>00000110011011111111000111101111011111111110001
Menjiagao1	000001100100100010000000101110000000100000000000001100100<br>00011101000010000000111010111100101000000001110
Xiaohonggu	000000000110011010000010111000000010000001100000001000000<br>01010001111100000000001100001000001001100000100
Jinxibai	0100000001111000100000010000000001101000000000000001101100<br>01001001000000111011110000000000001000110001100
Jinbaoyin	000000000111100010001000100000000001000000110000001100000<br>01101001000000000000011000001100010000000001000
Hongainuo	111110000011100010001000100000000110000000000000000100000<br>00001001110000011011110010010000101000000000110
Aizaizhan	111111111111110000000000100000000110100000000000000100100<br>01001001110000011011110000000000001010110001010
Honggu	000000000110011010000001111000000011000000110000000000000<br>11111000010000000000101000000000010000000000100
Zimi	000000000110000111010010111000000110000001100000001000000<br>11110111100000000000010000000000100000000000110

Name	Fingerprint Encoding
Tainong67	0000000000000101000010001000000001111000000010000000000111001000110000000000000100000000011000000001010
Xianggu	0000000000110011010000010111000000110000011000000001000011100011111000000000110000000100000000001000
Ximaxian	1111111111001000101101100000111100001111100111111111111000001100111111111110001110011110111111110001
Jinnante43B	00000000001111000000100101110000110100000000000000000000101110111100000000000010000010000000000000110
Xiangzaoxian7hao	00000000001100110000000101110000000100000000000000000000011010111000000000000011000010010110000000100
80B	0000000000111100011100010111000011010000011001100000000011111001100000000000010101001000010001100000110
Baoxie123B	0000000000000101000010001000000001101000000010000000100000111100000100000000000001000101000010000000010100
LimingB	1111111111001000101111110000111100001111100111111111111000101100100111111110001110011110011111111001
Jiangnongzao1hao	00000000011110001000100100000000011110000011000000000000111111011100000000000110011110000100000000110
Gu154	00000000000001010000100010000000011100000000100000000000010110000000000000000000100000100011000000000110
Ninghui21	000000000000010100001000100011110000111110011111110001100000100010010100000001011100111000000010111001
N 22	1111111111001000100011110000111100001111100111111111111000101100100111111110001110011110011111111001
Shuiyuan300li	1111111111001000101111110000111100001111100111111111111000001100100111111110010110011110111000111001
XiangaiB	00000000001100110100100101110001110100000110000000000001111000110000000000000100001000000000001000010
Aituogu151	00000000001111000000000011110000000100000011000000000000011010000000000000000101000110000010011000010 0
Taidongludao	11111111110010001000011100000000000010000000111111111110000011000111111111100011110111101110011100 01
menjiading2	000111111100100010110000101100001111000101100000000000011110001100000000000011100001001101110010000 01
Jiefangxian	0000000000111100000010010111000000010000001100000001001000100100110000000000000100001001011010000000100
Hongqi5hao	1111111001111000101101110000101100001111101111111111111000101100000111000010011001010110100111111001
Baikehualuo	00000000001100110100000101110000000110000011000000001000111010011001000000000001000010000110011000100 0
Liuyezhan	00000000001111000100000101110000111100000110000000100000101110011000010000000000000010000000001100001 00

Name	Fingerprint Encoding
Aihechi	00000000001111000100100010000001110100000000000010011001000100100001000001111100000000010100000001110
M202	00000000000000101111010100000000000101111110010000100001011101000011101111001011001001010100001011100
Xiangnuo	11111111110010001011011100001111000011111001111110111110000011001111111111000111101111011111111110001
Xuanenchangtan	00000000001100110100000101110000000100000011000000001000111010011111000000001010000100100100110001010
Jinzhinuo	00000000001100110000100101110000000100000011000100001000110100000011100000010011000000000010011000000 1
Laohongdao	11111111110010001011111100001111000011111001111111111110000011000111111111001111101111010111011000 1
Wuzidui	11111111110010001011011000001111000011111001111111111110000011000111111111000111001111011100011100 1
Xibaizhan	00000000001111001100000011110000000100000011000001000010011001111100010000100110000000100100111000000
Zegu	00000000001100110100000101110000110100000000000001000001110100110000100000011000001000010001100000000
Cungunuo	11111111110010001011111100001111000011111001111111111110000010001111111111001111001111001100011000 1
Lengshuigu	11111111110010001011011000001111000011111001111111111110001011000111111111000011101111000000011000 1
Banjiemang	00011000010010001011011001110000111000000110000000100000011100100000000000000000100101001011110000000 00
Dular	11111111110010001011111100001111000011111001110111111110001011001001111111100000100111101111101100 11
Wuzuhonggu	00000000001100110100000101110000000100000011000000010000001011001111100000000011000000001000011100011 00
Nanganggu	00000000001100010100100101110000000100000011000000010100001111001111100000000000100001000000000000011 000
Mowanggunei	11111111110001000000000011110000110100000000000001000000110011011000000000000110000010000000000000000 110
Qitougu	00000000001100110100000101110000000100000011000000010000001111001111100000000001100001111000010000010 01
Huangpinuo	11111111110010001011111100001111000011111001111111111110000011000111111111001111001111011100011000 1
ZS97	00000000001100110100000101110000110100000110011010000000011100111000000000010000101000010001100001 10
Nipponbare	11111111110010001011011000001111000011111001111111111110001011000000111111110011110011110111111111 001
MH63	11111111100001010000100011110000111100000000000000101000110000011000000000000110100100001000000000 1100

Name	Fingerprint Encoding
9311	0000000000000101000010001111000111010000011000000000000<br>111000000000000000000010000010000100000000000
Zhonghua11	1111111111001000101111110000111100001111100111111111111<br>000101100100011111110011111011110111000111001
Laoguangtou83	0000000000000101111011010001110001110000000000110000001<br>000001000111100111001011001001010000111011110
Balila	1111111111001000101111100000111100001111100111111111111<br>000101100100111111110001111011110111111111001
Nanjing11	0000000000000101000000010111000011100000011000000001000<br>011010011000000000000101000010000100000000100
2428	1111111111001000101101110000111100001111100111111111111<br>000001100100101111110001110011110011110111001
IRAT109	1111111111110011001101110111111100001111100111111011111<br>000001100111111111110101011011010111110111011
Heigeng2hao	1111111111001000101111100000111100001111100111111111111<br>000001100111111111110011110011110011111111011
Erjiunan1hao	0000000000110011010010010111000011100000011000000000011<br>011110011000000000000110000100001000110000101
ChaoyangyihaoB	0000000000110011010000001111000000001000001100000000000<br>011110011000000000000110000100101000110000101
L301B	0000000000000010010000010111000011110000011000001000000<br>111110011100000000000001010100001001110000110
Guangluai15	0000000000110011010000010111000011110000011000000001000<br>011110000000000000000101000010000100011000011 0
ZhuzhenB	0000000000110011010000010111000011010000011000000000000<br>011110011000000000000110000100001000110000001
Baimaodao	1111111111001000101111100000111100001111100111111111111<br>000101100011111111110001110011110111000111001
Baoxie-7B	0000000000110010010000110000111100001111101000000000000<br>001100000010000000001001100001001011000000001010
Gzhenshan97B	0000000000110011010000010111000011010000011000000000000<br>011110011000000000001110000100001000110000101
Nanxiongzaoyou	0000000000111100010000000100000000111100000000000010000000<br>111100011000100000000110000010000100000000000110
Zaoshuxianghei	0000000000110011010010001000000001111000000010000001000000<br>011000011100000000000010001000010000001000001 10
IR661-1	0000000000000101000010001000000001111000000010000001000000<br>001100011100010000000110001000010000000000011
Nanjing11	0000000000110011010000001111000000001000001100000000001000<br>011010011000010000000101000010010100000000000101
Gui630	0000000000000101000010001000000001110000000110000000000000<br>001010111000000000000100010010000100000000000111

Name	Fingerprint Encoding
76--1	000000000000010100001000100000000111100000011000000000000<br>0011001000000000000001000000100001010000000010
Xugunuo	000000000000010100001000100000000111000000011000000000000<br>0011001000000000000001000000100001010000000010
Taizhongzailai1	000000000011001101111000011100001110000001100000001000000<br>1111000110000000000001000001100011000001000000
AnnongwanjingB	111111111100100010111110000011110000111110011111111111111<br>0000011000111111111100011100111101110011100001
Xiangwanxian1hao	000000000011001101000000101110001001000000110000000000000<br>1001100000000000000001000000100011100110000110
Xingguo	111111111100100010111110000011110000111110011111111111111<br>0000011001111111111100111110111101111111111001
Zhonghua8hao	111111111100100010111111000011110000111110011111111111111<br>0001011001000111111100111100111101110001110001
Aimakang	000000000011110000000100011110000111100000011000000000000<br>1111100001000100000000000000100001000110000010
JWR221	000000000000010100001000111100011101000000110000000000000<br>1110000000000000000001000000100101000000100000
Zhengxian232	000000000011001101000000111100000000101110000000000001000<br>0111100110000000000001100001000010000000000100
Chenwan3hao	010000000011100010000001001100001101000000000000001000000<br>1110100000000000000000001001000010001110000000
Maguzi	111111111100100010110010000011110001111110011111111111111<br>0000011001001111111100011110111101111101110001
Aimi	111111111111111000011100101110000110100000011000000000000<br>0100100110000000000101100001000010100000000100
Haobayong1	111111111100100010110111000011110000111110011111111111111<br>0001011001111111111100001110111101110001110001
Ajiaonante	111111111100100010111111000011110000111110011111111111111<br>0001011000111111111100011100111100111111111011
Gallawa	000000000011001001110111000011100011011111010011011000<br>1<br>0111010000111100000000110011010101101100110100
Karayal	000000000011001001110110100011100011011111010011011000<br>1<br>0111011000111011110000110001010101000000011110
Srav Prapay	010000000011110011000001000000000000100000000000000100010<br>1100000111111000000000100100000000000000110011000
Nang Bang Bentre	000000000000001011110110100011100000000000000000100100000<br>0111010000111001111010110010110100000110101100
DJ 24	000000000000001011110110100011100000000000000000100001000<br>0110010000111001110010110010000101001110100000
DJ 102	000000000000001011110110100011100001000000101001001010110<br>0111110001111001111010110010010101001010100000

Name	Fingerprint Encoding
Santhi 990	000000000011110001110110110011100001011101001011001000 1<br>00000100011111000010101101001101010000001010 1
UZ ROS 7-13	0000010000101100011101101000111000100000110000100110000<br>0010100000111000001010110010010001100010111 00
SL 22-620	0000000000110010111101101000111000101110110110110110001<br>0011010000111111000000111010110101000001100 00
Spin Mere	1111110000110010011101101000111000100111110100100110000<br>0010100000111000001010110000010101100010111 10
AMANE	0000000000111100111100010000100011010000000000011101000<br>1001100001000000000000110000100000100000011 110
Padi Tarab Arab	1111111111110011000001110000111100001111100111111011011<br>0001011000111111111100011110111101111111110 11
P 35	0000000000110010011101101000100000011111110100110110001<br>0110100000111001110000110010010101000010101 10
CAROLINO 164	0000010000000010111101101000111000000000000000100101010<br>0111110001111001110010110010110101000100100 00
HKG 98	0000000000110010011101101000111000000111111001011011000 1<br>0111010000111111110000111110110101001110100 10
Daudzai Field Mix	1111111111110010011101101000100000110000101000110110011<br>0110110000111000000001011000001010100000010 010
JP 5	1111111111000010110000001111111000100000110100011100000<br>0101100000110000000010110011000011001100101 00
Hi Muke	1111110000110010011101101000111000110001110100100110000<br>0010100000111000001000110000010101100010101 10
WIR 911	1111111111001000101111110000111100001111100111111111111<br>0001011000111111111100111110111100110011100 01
Lua Chua Chan	1111111111001000101101110000111100001111100111111011111<br>0001011001111111111100110110111101111111110 11
Sereno	1111111111111100000000001111000011110000000000001000000<br>1000110101000000000001100000100100000000000 000
ARC 10633	1111111111000100010000001111000000000000000000000101000<br>1000110111000000000001110000100001100000001 000
Simpor	1111111111001000101100110000111100001111100101111011111<br>0000011001111111111100010110111101011111100 01
Heo Trang	0000000000110011010000001111000000001000000000000101000 00<br>1100100011000000000001110000100000000001000 1100
THAVALU	0000000000110010011101101000111000110111111010011011000 1<br>0111011000111000000001011001001010100000010 110
WC 10253	1111111111110011010001110111111110000111110011111101011 1<br>0001011001111111111101010110111101111111110 01
KRASNODARSKIJ 3352	1111111111001000101111100000111100001111100111111111111<br>0011011001001111111110010110011110011111111 011

Name	Fingerprint Encoding
EMBRAPA 1200	1111111111001000101111110000111100001111100111101011111<br>000001100111111111110101111011110111001110001
WAB462-10-3-1	0000000111110011001101110111111100001111100111110010111<br>010001100111111111110101011011110111001111001
Bombilla	1111111111001000101111100000111100001111100111111011111<br>000101100011111111110001111011110111111111001
TCHAMPA	0000000000000010111101010000000000101111000010100100000<br>010101101011100000001011001011010100001011000
BHIM DHAN	1110000000110011001111101000111100001111100111111011111<br>000001100111111111101001011101111011111110001
WC 3532	0000000111110011010001110111111100001111100111111010111<br>000001100111111111110101111011110111111111001
Kin Shan Zim	0000011111111110000000001011100011101000001100000000000<br>101110011000000000000000000100000000000000100
Yong Chal Byo	0000000000110001000010010000010100001111100111111111100<br>000010100011011111110000001100110000110000000
Pan Ju	0000000000110011010000001111000011010000011000000100000<br>011110011000100000000100000010000100011000000 0
Buphopa	1111111111001000101101110000111100011111100111111111111<br>000001000011111111110000110011110111000110001
TAINO 38	1111111111000000110001110111111100001111100111101111111<br>110001100111101111110101011011110101001110001
2	1111111111001000101111101000111100001111100111111011100<br>001001100011111111110101110011110101111111001
LUSITANO	1111111111001000101111100000111100001111100111111111111<br>000101100111111111110011111011110111001111001
IARI 6621	0000000000110010000000001000000000010111101101000010100 0<br>011101111011100111001011001011010100000000100
Hsin Hsing Pai Ku	0000000000111100000000001011100001101000000000000110000 00<br>111110000011010000000100000010000100000000000010
TD 70	0000000000110011010000010000000000001000000000000000100100<br>000010011000000000000100010010010100000000000100
Dara	0000000000000010111101010000100000101111010110101100000<br>010101000011101111001011001011010100001010000
Shimla Early	1111111111111001101000000011100001101000000001000001110 00<br>111101000100000000000111000010000001000111100 00
LA PLATA GENA F.A.	1111111111001000101111101000000001110000000001111100000 00<br>001001000011011111110101110001100111001011000
CM1, HAIPONG	0000000001111100100010001000000000110000001100000000000 0<br>110001010100100000000101011010000100011000000 0
4595	0000001000110011010000010111000011110000001100000000000 0<br>111010000100000000000001000010000100011001100 0

Name	Fingerprint Encoding
A 5	1111111111001000101111100000111100001111100111111111111<br>000001100111111111110010110011110111111111001
Ittikulama	0000000000110010011101101000111000100111110100110110001<br>011101100011101111000011000101010100011010110
DNJ 179	0000011110101100011101001000111000010000000000100101010<br>011101000111100111101011001010010100001011000
DNJ 121	0000000000000010111101001000111000110000011000100101010<br>010001000011100111001011001011110100001010100
Jyanak	1111111111001000101100101000111100010111110111111011111<br>000001100111111111110100110011110101111110001
Ao Chiu 2 Hao	0000000000110011000010010111000100010000011000000100000<br>011110011100000000000100000010000000000001000101
GUYANE 1	0000000000111100110000001111000011010000000000011100000<br>011111011000000000000110000010000110011000001 10
10340	0000000000110011010000010111000100000000000000000100100<br>010110011011110000000100000010000100011000010 0
BR11	0000000000000101000010001000000011110000000000000000000<br>101000011000000000000100000010001000000000000100
BR24	0000000000111100000001000111100000011000000000000100000<br>111111011000000000000110000000000110000100111 0
93072	0000000000111100000001000011100001101000000110000000000<br>101000000100000000000010100001000010000000001 10
108S	1111111111000010100001000111100001101000000110000010000 11<br>111100000010000000000001100000110011000000000010
C418	0000000000111100000001000011100001110000000110000000000<br>001000000100000000000011100001101110100000000110
CDR22	1111111111000010100001000111100001111000000000000000101000<br>101100011000000000000111010010000100000000101 0
Chenghui448	1111111111001000100010001101000011110000000001000010000 0<br>110100011000000000000011010010000100000010010 10
Fengaizhan	0000000000000101000000000100000000111100000011000000000000<br>111100011000000000000100000001100010000000000110
Gang46B	0000000000000101000000000100000000010100000011000100000000<br>111100000100000000000010000101000010000000000100
Gumei2hao	0000000000110011000000000100000000110000000001000000000000<br>101100000000000000000111001010000100000100000100
Huajingxian74	0000000000000001000010001000111100001111100000000000000 0<br>001100000000000000000011000001000010000010000100
M3122	0000000000000101001110001000000000100111110011111111001 11<br>100101100101110000000101110011110000000111001
R644	0000000000000101000010001000000001111000000010000000000000<br>000100111000000000000000000001100110000000000100

Name	Fingerprint Encoding
Shennong265	000000000000100010001010000011110000111110011111111111<br>00000110010011111011000111001111011111111001
Y134	000000000000101000000000101100001111000000100000000000<br>10001010000000000000001000001100100000000001000
Yuanjing7	000000000000100010001010000011110000111110011111111111<br>0001011000000111101100011100111100111111001001
Yuexiangzhan	111000000000010101110001000000001111000000110000000000000<br>0011000000000000000000101010010000110000000110
Zaoxian14	000000000011001101001001011100001111000000100000001011<br>1111100110000000000000110001100001000110000100
Zhong413	000000000000101000010001000000001110000000110000000000000<br>011100100000000000000010000000100001000000001010
Zhongyouzao 81	000000000000101000010001000000001110000000110000000000000<br>01001001110000000000001000000100001000110000110
Zihui100	000000000000101000010001000000001111000000110000000000000<br>01001001110000000000001000000100001000110000000
Bg90-2	1111111110000101000010001000000000001000000010000000000000<br>110000000011000000000010000110000000000000000010
IR72	1111111111000100011100001111110000010111011000000100000<br>1000000111000000000001100000100001000000000000
PR106	000000000000101000010001000000001111000000010000000000000<br>10110001100000000000001000011000110000000000010
TKM9	000000000000110100001000100000000110111100000000000110000<br>111111010100000000000011000000000110000000110000
Amo13 (Sana)	0000000000000101000000100100000000001100000011000000101000<br>1111000110000000000000010000000000010000000000010
Khazar	1111111111001000110001110000111100001111101000010011111<br>0011011000111111111100000011011100111001110001
Gayabyeo	1111111111001000101110001000000001111000000010000000000011<br>1000000000000000000000010000000000010000000000000
Iksan438	1111111111001000101101100000111100001111100100000100010<br>11110010000000100000000100010011101101000000000
Milyang23	0000000000000101000010001000000001111000000010000000000011<br>100100011000000000000001000000000110000000001110
MR185	0000000000000101000010001000000001111000000010000000000011<br>100100011000000000000110000000000110000000001110
Manawthukha	1111111111111110011000000100000000001000000010000000000000<br>111100011111000000001100100000000100000000000010
Shwe Thwe Yin Hyv	111111111100010101111000011100000000101110110000000100000<br>100000011100000000000001000011000100000000000100
Bg300	0000000000000100000001000100000001001100000001000000000000<br>1100100000110000000001000000110001000110000010

Name	Fingerprint Encoding
Bg94-1	11111111100001010000100010000000100100000000100000000000<br>1100100000110000000010001001001000000000000110
CR203	00000000001111000110100100000000111100000000000000000000<br>1000001110000000000000100000100001100000000000
OM997	00000000001101000111100001110001001000000000000000000000<br>1000000111110000000000100000000001000000000000
PSB RC 28	00000000000001010000100010110000000101110100000000000000<br>1000000000000000000000100000000000100000000100
PSB RC 66	11111111010010001011000011110000111100000000000000100000<br>1000000111000000000011101001000010000001000000
TEQING	00000000000001010000100010000000111100000110000000000000<br>0111000110000000000001000000100001000000001010
IR68552-55-3-2	00000000000001010000100010000000111000000110000000000000<br>0111001000000000000001000000100001000000001110
IR66897B	00000000000001010000010011110001111000000110000000100000<br>1011000000000000000001101000000010000000001100
IR58025B	00000000000001010000100010000000000101110110000000000000<br>1100101000110000000010000001000110000000000010
Dhan4	11111011010010000011111000001111000011111001101110111111<br>0001010001001101101100111100011101111111110101
Mayang Khang	00000010001100110100000101110000111000000110000000000000<br>0011100010000000000010011001001000000110000100
E B Gopher	11111111111110011010001110000111100001111100111111011111<br>0001011000111111111010111101111011111111110001
C 5560	11111111110010001011011100001111000111111001111111111111<br>0000010001111111111100000110111101110001100001
Quinimpol	11111111111110011001101110000111100001111100111111011111<br>0000011001111111111100011110111101111110111001
TAICHU MOCHI 59	11111111110010001011001100001111000011111001111111011111<br>0000011000111111111100000110111101111110110001
WC 2811	11111111111110011010001110111111100001111100111111011111<br>0000011001111111111101010110111100111111110011
Criollo Chivacoa 2	11111111111110011010001110000111100001111100111111010111<br>0000011001111111111100010110111101111111111001
Secano do Brazil	11111111111110001010001110111111100001111100111111011111<br>0000011001111111111100111110111101111111110011
BERLIN	11111111111111000000000001111000011110000000000000001000000<br>1000110101000000000000100000100100000000000010
Sel. No. 388	11111111111110011010001110111111100001111100111111111111<br>0000011001111111111101010110111100111111111001
Pusa (Basmatil)	00000000000001000111011010000000100010000000101111110000<br>0010101110000000000001101101011100000000111000

Name	Fingerprint Encoding
ASD16	00000000000001010111011010000001001100000010111111100000<br>001010111000000000000101101011100000000111000
Dianjing	11111111110010001000011101111111000011111001111111111111<br>000001100100111111110101110011110111110111001
F6	00000000000001010000100010000000110100000010000000001000<br>100010011100000000000011000000000110000000000
452	00000000000001010000100010000000111100000010000000000000<br>110000000000000110001100000000001000000001000
NAN-29-2	00100010000000000111011010000001000000000010111111110100<br>001011111000000000100001101011100000000111000
Ai-Zi-DAO	11111111110001000111100010000000000101110110000000100000<br>111000101000000000000110000010010110000000000
Babaomi	00000000001100110100000011110000000100000011000000000000<br>011010011011000000001010000100001100000000101
Diantun502	00000000001100110100000101110000110100000011000000000000<br>110110000000000000000110101000000100000000100
Heiheaihui	11111111110001000000100101110001111000000000000000100000<br>111000011000000000000111010110000100000000010
Jiangxisimiao	11111111110001000000100101110001111000000000000000100000<br>111000011000000000000111010110000100000000110
Laohudao	11111111110001000010100101110001111000000000000000100000<br>111000011000000000000111010110000100000000110
Linjintangdao	00000000001111000000000100000000111100000000000001100000<br>110010010000000000000110000010000100000000010
Mengguanda magu	00000000001100110000000101110000110100000011000000000000<br>111010001100000000001010000100000000110000100
Pengshantiega nzhan	00000000001100110000000101110000110100000011000000000000<br>111010011100000000001010000100000000110000110
Wumanggaon uo	00000000001100110100000101110000110100000011000000000000<br>110110000000000000000111010100000100000000110
Yunguang8ha o	11111111110010001011011000001111000011111001111111111111<br>000001100000111111110001111011110111111111001
Zhongchao12 3	11111111110010001011011000000000000001111100111111100111<br>000001100100111111110101110011110000001110111
Gizal159	11111111111110011010001100000111000001111100111111011111<br>000001100000111111110001110011110011111111001
Khao Daeng	00000000001111001000000101110000111100000011000000000000<br>111110011000000000000110000100000101110000101
Basmati370	11111111110001010111100001110000000101110110000000100000<br>100000101000000000000100001100010000000000000
Bhavani	00000000001100110100100101110000111000000110000000100000<br>001010011000010000000100100100001000000001110

Name	Fingerprint Encoding
IR50	000000000011110000000100011110001111100001000000000100100<br>111000000001100000000011010000000100000000001110
Jhona349	0000000000000010111101110000100000101111110110100100000<br>0011011000111011110010110010010101000000011010
Karnal Local	0000000000000010111101110000100000101111110110100100000<br>0011011000111011110010110010010101000000011000
Type3	0000000000000010100001000100000000111100000001000000000011<br>1011000110000000000000100000100011000000000010
IRAT352	111111111000010100000000011110000000010000100000010100100<br>101000000000000000000011010110010000000110001010
TB154E-TB-2	000001111000010100000000011110000000010000100000010101100<br>1010000000000000000001110101100101000001100010
Domsiah	00000000000000101111111000000000110111111001111111010100<br>0000011000110111111110101111011100111001111001
Tarommolai1	00000000000000101111111000001111010011111100111111010100<br>0001011000110111111110101111011110101001111001
MR77 (seberang)	1111111111111100110000000100000000010000000010000000000000<br>11110001111110000000011001010000010000000000000
ir6	0000000000000101000010001000000001111000000010000100000000<br>1010001000001000000001100000100001000000100000010
At354	00000000000000101000010001000000000110000000100000000000000<br>10000000000000000000011010000000000000111000110
BG304	0000000001000101011110010000000000001011101100000000000000<br>10001000000110000000001100000110010000011000000000
Lemont	1111111111001000110001110111111110000111110011110101010111<br>0000011000111111111101010110111101110011100001
M401	1111111111001000101111111000011110000111111001111111110111<br>0001011001001111111110100110011110011110110011
OM1706	0000000000111100000001000100000000111100000001000000010000<br>1000001110000000000000100000100001000000000100
OM1723	0000000000111100000001000100011000011000000010000000100100<br>1000001110000000000000100000100001100000000100
X21	111111111110001000111100011111100001100000001000000010000<br>1000001110000000000000100000100001000000000100
X22	111111111110001000111100001111110000100000001100000000000<br>1010000110000000000000110000100000100000000001100
X23	111111111110001000111100011111100111000000011000000000000<br>1011000110000000000001100000100001000000000001100
C71	11111100000001000101111110000000011100011101001111111111111<br>00000001000001100000000101110011100001111111101
C70	00000000000000101000000000100000000111000000011000000000000<br>0111000110000000000001000000100001000000000110

Name	Fingerprint Encoding
Q5	00000000000001010000000001000000011110000001100000000000<br>0111000110000000000001000000100001000000000110
Suiyangnian	00000000001100100100000101110000000100000011000000100000<br>0110100111000000000001010000100001100001001100
Pokhreli	11111111100001010000000001111000011110000000000000101000<br>1110010110111011110000101000000001000000111010
Govnd	00000000000001010000100010000000111100000010000000000000<br>1011000110110000000001000111100001000000000110
UPR191-66	00000000000001010000100010000000111100000010000000000000<br>1010100110110000000001000011000110000000000010
ASD18	11111111100001010000100011110000001100000000000000000000<br>1111100000000000000001100000000111001100011000
TGMS29	00000000000111000000100011110001111100000110000001100000<br>0111000000000000000001100110100001000110000101
Phalguna	00000000000001010000100010000000110100000110000000010000<br>1010000000000000000001001010000011000000000010
Ajaya	00000000000001010000100010000000001100000010000000000000<br>1000110110000000000001000010000000000000000010
Dodda	11111111100001010000000001111000011110000000000000101000<br>1110010110111011110000101000110010000000111010
Palung 2	11111111100001010000000001111000011110000000000000101000<br>1110010110111011110010101000110010000000111010
TKM6	11111111111100010100011101110000111100000001000000100011<br>1011100110110000000011001110001010000000001100
RUSTYLATE/中413	11111111111110001000000010000000110100000000000101100000<br>0110100100000000000100100100100001000000001000
Yenfangghu	00000000001111001000000101110000111100000110000000001000<br>1111100110000000000001110000100000100110000101
Heimichut	00000000001100110000000101110000000100000011000000000000<br>0110101111000000000001010000000000100000000100
Teksichut	11111111100100010111110000011110000111110011111011111<br>000001100111111111110011110111101111111111000
Eiko	11111111100100010111110000011110000111110011111011111<br>000001100111111111110011110111101111111111001
RUBIO	11111111111100110100011101111111000011111001111110111111<br>0000011001111111111100011110111101111111110001
SADRI RICE 1	00000000000000101111111000000000110011111001111110101011<br>0001011000110111111101011110111101010011110001
Sereendan Kuning	11111111111100110100011101111111000011111001111110101111<br>0000011001111110111101010110111100111111110011
SAI-BUI-BAO	11111111111100110100011101011111000011111001111110101111<br>0000011001111111111101010110111100111111110011

Name	Fingerprint Encoding
ZIRI	000000000000001011111110000000001100111110011111010101<br>0001011000110111111100011110111101010111111001
Dacca6	000000000000001010111110000000001101111110011111010111<br>0000011000110111111101011110111001110111111001
Latisai1	000000011111001100000111000011110000111110011110101010100<br>0000010001111111111101010110111100111111110001
Amareles	000000000000001010111110000000001101111110011111010111<br>0000011000110111111101011110111001110011111001
Up15	111111111000010100000000111100001111000000000000000101000<br>1110010110111011110000101001000010000000111010
Uz-Rosz 275	111111111111001101000111011111111000011111001011110111111<br>0000011001111111111110101111011110011111111001
Ginga	111110000011001101111110100001100000111110001110101010100<br>0000001000110011110101111100110101101111111101
Bintapan	000000000110011010000010111000111100000001100000000001000<br>0111101110000000000101010000100001000110000100
SADAJIRA-19-<br>303	000011111000010100001000111100001111000000010000000100000<br>1101000111110100000001101010000010000001001110
CHOROFA	000000000110011010000000111100001101000000010000000000000<br>1100100000000000000010000000100011100110000100
La110	000011111000010100001000111100001111000000010000000100000<br>0101000111110100000001101010000010000001001110
Yunjiang35	111111111000010100000000010000000011100000000100000000100000<br>1010100000000000000100100100110001100000001010
Yunhui72	000000000000010100000000010110000111000000011000000000000<br>111000000010000000000001100001000011000000001010
Wudadaozhon<br>g	000000000000000101001000100000000111100000011000000000000<br>01000000000000000000010000000100011000110000100
IR65600-27-<br>1-2-2	000000000110011001110001111110011110000000000000000100000<br>1111101110000000000001000100100001100000000101
Yetuozai	000000000000010100000000010110000111100000011000000000000<br>0111001111000000000001100000100001000000001010
P59279	000000000000011001001001011110001101000000010010010000000<br>1111000110000000000001100000100001000000000100
Tsao wan<br>ching	000000000011110001001000100000000110100000000000000001000100<br>10001001000010000001101100000000001010010001110
Anambae<br>ndanggalasi	111111111111100110100011100001111000011111001111110111111<br>0000011000111111111100111110111101111111110001
Er chiu ching	000000000011110010000000101110000110100000011000000001000<br>0111100110000000000001100000100001000110000110
Heen goda<br>wee	111111111111100110100011100001111000011111001111110101111<br>0000011001111110011101011110111100111111111001

Name	Fingerprint Encoding
Pathma wee	00000000001100100111010110001000110100000000000011001000<br>1101100001110000000000100100100001100001001000
Lakkhi kajal	00000111101011000111011010001110000100000000000110100000<br>0101110001111001111010110010110100001110110101
Moisdol	00000000000000101111011010001110000100000000000100000000<br>0101010001111001111010110010010100001110100101
Jabor sail	00000000000000101111011010001110001100000000000100100000<br>0000010000111001110010110010100101001110110001
Sada solay	00000000000000101111011010001110001000001101100101100000<br>0101100000111001100010110000010101001000101001
Sholay	11111111111001000101111110000011110000111110011111101111<br>0010011000000111000110011111011010111111111100
Ma ba you zhan	00000000001111000000100100000000110100000000000001100000<br>0100100111000000000101100000000000100011000011
Daegujo	11111111111001000101111110000011110000111110011111111111<br>0001011001111111101100011110111100111111111100
Paikasa	11111111111110011000000110000111100001111100111111011011<br>0000011001111111111110101111011110111111111101
Uwi	11111111111110011001101110000111100001111100111111010011<br>0000011000111111111100001100111101011111100011
Garia	00000111101011000111011010000110001100001100001101000000<br>0111110000111000000001011001000010100001011000
AUS 449	00000000000000101111010100001110000011100000000100101000<br>0000010000111001110010100010100101000010101101
Dourado Precoce	00000001111100110000001101111111000011111001111110111111<br>0000011001111111111110101011011110111111111100
IR 661-1-140-3-117	00000000000000101000010001000000001111000000100000000000<br>1011000110000000000000100000100011000000000110
Aichi Asahi	11111111111001000101101100000111100001111100111111111111<br>0001011001001111111110001110011110011000111001
SHIMIZU MOCHI	11111111111001000101111110000011110000111110011111111111<br>0000011000001111111110001111011110011111110011
KRASNODARSKIJ 424	11111111111001000101111110000011110000111110011111111111<br>0001011001001111111110010110011110011111111011
Pergonil 15	11111111111001000101111110000011110000111110011111111111<br>0001011000111111111110001111011110111001111001
Red Khosha Cerma	11111111111001000101111110000011110000111110011111000100<br>0000011110110011111100011100111001010011101000
Safut Khosha	00000000001100110111010100001110001011111010001011000000<br>0001001000111000000001011001001010100111010100
NORIN 11	11111111111001000101111110000011110000111110011111111111<br>0000011000111111111110011111011110111111111101

Name	Fingerprint Encoding
R 75	1111111111110011010001101000111100001111100111111011111<br>00000110011111111111100010110111100111111110011
UZ ROSZ M38	1111111111001000101100110000111100001111100111111111111<br>0001011000111111111100001100111101110011100011
H57-3-1	1111111111110011010001110111111100001111100111111110111<br>00000110011111111111001111101111011111111111011
IARI 6626	0000000000110010011100010000000000101111110110000101000<br>0011011110111001110010110000010101001110101000
IR 2071-625-1-252	1111111111000100011100001011000000010111011000000100000<br>100000111000000000000101000110001100000000000
Khoia boro	1111110000110010011101101000111000100111110100100110000<br>0010100000111000001000110000010101100010111110
IR 8	0000000000000101000010001000000000010000000100000000000<br>0011000000000000000000100000000001100000000000010
LABELLE	1111111111110011010001110111111100001111100100011000100<br>00000111100010111111010101101100110011100000001
LEBONNET	0000000000110100011110000111000100100000000000000100100<br>1010000011110000000001000000000001000010000100
AUS 371	0000000000000000111101010000111000100000000000100000000<br>00010111101110111100101000101101010000101010100
AUS 373	0000000000000010111101010000111000101000000000000000000<br>00000111111110000000101100101111010000000101000
IR 36	0000000000000101000000000101100000000101110110000000100000<br>100000111000000000000101000110001100000000000
JAYA	1111100000110010011100010111000011010000000000000000000<br>100010000000000000000011000010000000100110000110
NEW BONNET	1111111111001000101111110000111100001111100111111111111<br>000001100011111111110101011011110111001110001
IR 64	0000000000110100011110000111000100100000000000000000000<br>1010000111110000000001100000000000100000000011000
KUNTLAN	1111111111001000110001100000111100001111000111111011111<br>0001011000000110010100001110110101111111000011
British Honduras Creole	1111111111110011010001110111111100001111100111111011111<br>00000110011111111111010101101111001111111110001
CPSLO 17	1111000000100110000000001011111111000011111001111110000011<br>11000101101111000000011001101001011100111000 1
AKITAKOMACHI	1111111111001000101111110000111100001111100111111111111<br>00010110000011111111001111001111001111011110001
BASMATI 385	0000000000001000101111110111000000001111110010000101000 0<br>0010011000110111111110101111011100100001111000
AGNO (PSBRC28)	0000000000000101000010001011000000001011101000000000000000<br>1000000000000000000001100000000000100000000000000

Name	Fingerprint Encoding
PSBRC82	0000000000111100000000000011100001111000000010000000000001000001110000000000000100000100001100000000000
NSICRC122	0000000000000101000001001000000011110000100000000000000101100001100000000000111000011100100000001000100
BRRI DHAN 29	1111111110000101000010001000000000001000000010000000000001100000000110000000000100000000000110000000000010
PR 116	0000000000000101000000000100000001111000000010000001000001011000000000000000000001000000100101000000001000110
CIGEULIS	1111111110000101000010000111101111000000000000000100000101000011111000000000110010000000010000000000100
OM 2517	0000000000000101000000000111111000010000000100000000000001010001010000000000000111000010000000000000001100
BRRI DHAN 28	0000000000000101010010001000000001111000000110000001000001000000110110000000000100000000001000000000001100
CIBOGO	1111111110000101000010000111100100100000000000000100000101000011111000000000110010000000010000000001100
NEDA	0000000000000101000001100000000000010000000110000001000001010000110000000000000100001111001000000001100
CIHERANG	1111111110000101000010000111101111000000000000000100000101000011111000000000110000000000010000000000100
Qiuqianbai	0000000000110011000000000110100010010000000110000011001001111100010000100000011011001001000000000000000
Guangkexiangnuo	111111111100100010111111000011110000111110011111111111000001100001111111110011110011110111000111001
Wenxiangnuo	1111100000111000100000000111100000001100000110000000100000100000011000100000000111100100010100000000000100
Laozaogu	0000011111111110000000000001111000011010000000000000010000001100110111000000000001100000100100000000000000010
Lengshuinuo	0000000000000101000000101011100001101000000010011000001000011100110000000000001000000100001000000000110
Feienuo2	111111111100100010110111000011110001111110011111111111000001100111111111110000110011110011000110001
Zimangfeie	111111111100100010110111000011110000111110011111111111000001100011111111110010111011110111000110011
Guantuibaihe	111111111100100010111111000011110000111110011111111111000001100101111110110001111011110111111110101
Haolvguangnian	111111111100100010110111000011110000111110011111111111000001100011111111110010111011110111001110011
JinghuB	111111111100100010111010000011110000111110011111111111000101100011111110110011110011110011111111001
Sibeitichao6	111111111100100010110000100000000010001000011111010001000110010010000000000010111110111011111111111001

Name	Fingerprint Encoding
GPNO 5055	0000000000111100000000000111111110000111110011110101111<br>1000001000111110000000101111101110011111100100 1
ASWINA 330	0000000000000010111101101000111000110000011000110110011<br>01101000001100000000101100100100010011101001 0
Khao Luang	1111111111001000101101110111111100011111100111111111111<br>0000011001111111111100001100111100110001100 01
C 8429	1111111111111001100110011011111111000011111001111110111111<br>0000011000111111111100011110111101111111110001
Warrangal Culture 1252	0000000000111100000000000111100001101000000000000000001000<br>100010011000010000000100010010000110000000111 1
Padi Pohon Batu	1111111111111001100110111000011110000111110011111101101 1<br>0001011001111111111100011110111101111111110011
NC 1/536	0000000000000010111101101000111000110000101100110100000<br>00000100001110111100101000100101010000001110 0
Won Son Zo No. 11	0000000000110011010000010111000011010000000000000000000<br>111110000000000000000100000010010100100100010 0
Chacareiro Uruguay	1111111111001000101111100111111100001111100111111111111<br>0000011001111111111100011110111100110011110 01
Doble Carolina	0000000000000010111101110000011000110000101100001100000<br>00100100001110111100101100000101010000001110 0
Ai Chueh Ta Pai Ku	0000000000110011000000010111000011010000000000000001100<br>01101001110000000000001001000100000011100001 11
Thang 10	0001100001001000100000010111011000010000000100000010000 0<br>01011101010010000000111010000011100111000011 0
Sipirasikkam	0000000000110011000010010111000000001000001100001000000 0<br>10111000000000000000011000001000000010110000100
TJ	0000000000110011000010010111000000001000001100001000000 0<br>1011100000000000000001000001000000010110000100
PD 46	0000000000111100100000001111000011010000011000000000000 0<br>11001000000110000000000100001000010000000001 00
PATNAI 6	0000000000000010011101010000100000111110000010101100000<br>01010111101110011100101100100101010001101000 0
K8C-263-3	1001111111000010100010010111000000001000000000001001001 00<br>01011001010000000000110010010001100000000000 0
Chun 118-33	0000000000110011010000010111000111000000001100000001000 00<br>01111001100000000000010000010000000000010011 00
Manga Kely 694	0000000000000010110000000111111100011000001010000000001 00<br>01011010011110000000101000110100101100100110 0
BLUE STICK	1111111111001000101101100000111100001111100111111111111 1<br>0001011000111111111100011100111100111111110 01
Nam Dawk Mai	0000000000111100000001000111100001110000000110000000000 00<br>11111000010001000000010000010000010011000011 0

Name	Fingerprint Encoding
INIAP 7	0000000000111100010000001000000000100000001000010000010<br>101000011000100000000100000100000000001000110
Onu B	1111111111001000101101110111111100001111100111111011111<br>000001100011111111110001011011110011000110011
Red	0000000000000010111101101111111000100000110100100011000<br>010010000100000000001010001100011101000111110
Dichroa Alef Uslkij	1111111111001100100010001000000011110000001000000000000<br>110000000011000000000110001000001000000000110
BKN 6987-68-14	0000000000000101011110000111110100100000001000000000000<br>111000010000000000100100000100000000000001100
KUBANETS 508	1111111111001000101111100000111100001111100111111011111<br>000001100000111111110011111011110111001110001
IR 9660-48-1-1-2	0000000000000101000010001000000000101111100000000000000<br>100100000000000000000100000100011000000000010
Jumli dhan	1000000000110011001111110000111100001111100111111010111<br>001101100011111111010011111011110111011111001
N-2703	0000000000110010011101101000111000101111000000101100000<br>011001111011101111001010001011010100011000000
PHUDUGEY	0000000000110010111101110000110000101111010110010100000<br>010011000011100111001011001111010100000011110
Ak Tokhum	0000000000000010101111110000011111101111110011111110011<br>000001100011011111110011111011100111011111001
RP2151-173-1-8	0000000000000101000010001000111000100000101000000000110<br>101000000000000000000100001000001100000000010
HB-6-2	1111111111001000101111110000111100001111100111111111111<br>000101100111111110110011111011110111111111001
GPNO 1106	1111111111110011011001110111111100001111100101111011111<br>000001100111111111110101011011110011111110011
Toga	0000000000111100000000001111000000001000000000000100000<br>111010000000000000000100100000000110100000 1100
Ragasu	1111111111001000101100110000000100001111100100101011100<br>000001011000000111110001101011110000111011001
Tamanishiki	1111111111001000101111100000111100001111100111111111111<br>000101100000111111110001110011110011111111001
Grassy	1111111111110011010001110111111100001111100111111011111<br>000011100111111111110101011011110011111110011
Kao Chio Lin Chou	0000000000110011000010010000100011110000000000000100100<br>001110011000010000000000000100001000110000100
Niwahutaw Mochi	1111111111001000101111100000111100001111100111111111111<br>000101100100111111110011111011110111111111001
Somewake	1111111111001000101111100000111100001111100111111111111<br>000101100011111111110011111011110111001111001

Name	Fingerprint Encoding
Ardito	11111111110010001011111000001111000011111001111111011111<br>000001100011111111101111100111101110011111001
NANTON NO. 131	11111111111100110100001100001110110011110001111111011111<br>000101100011111000010000011011110111111110001
Vary Tarva Osla	11111111110010001011111000001111000011111001111111011111<br>000001100111111111101110100111101110011110001
CSORNUJ	11111111110010001011111000001111000011111001111111111111<br>000001100011111111100111110111101110011111001
R 67	11111111111100110100011101111111100001111100111111010111<br>000001100111111111101010110111110011111110001
IR 238	11110000001001100000000101111000011110000000000000100011<br>110001010011100000000110001000010100010110000
Mitak	11111111111100110100011101111111100001111100111111011111<br>000101100111111111110001111011110111111110001
Gazan	11111111111100101011111000001111000011111001111111111111<br>001001100000111111110011111011110111111110001
99216	00000000001100110111010100001110001011110000000100000000<br>000001111011101111001011001011010100001010000
Shui Ya Jien	00011000010010001000100010000000000110000011000000000000<br>100000010000000000000110000010000100000000110
AKP 4	11111111110001000000000011110000000100000000000011000000<br>110110011000110000001110101000000000000000100
SORNAVARI	00000000000000100111010100001000001011110101101001000000<br>010001000011000111001011001001110100001011100
IR 2061-214-2-3	00000000001111001000100001111100001100000110000000100000<br>101000011000000000000100001100000000000001100
TAINUNG 45	00000000000000100000010001111000011110000000000001001000<br>110011011100000000000100100100001001111000110
Sapundali Local	00000000001111001100000001110000110111110100000111110000<br>111111000100000000000111000100001100000111000
Tauli	00000000000000101111011010001110000000000000000100000000<br>011101000011100111001011001000010100111010000
79	00000000000010001011111000000000110111111001111111010100<br>001001100011011111110101111011100100001111001
B805D-MR-16-8-3	11111111100001010000100010000000000010000011000100000000<br>100100100011000000001110101000000100011000110
A 152	00000000001100110110011010000000100110000011011111010000<br>000011100111110000000001100011010111001110111
UZ ROS 59	00000000001100110100000101110000111000000011000000000000<br>111110011000000000000010000100000000000000100
Gasym Hany	01000000000000101011111000000000110111111001111111010110<br>000001100011011111110101110011100111001111001

Name	Fingerprint Encoding
Celiaj	1111111111001000101111100000111100001111100111111111111<br>0000011001001111111100111110111101111111111011
CNTLR80076-44-1-1-1	0000000000111100001010001111000100010111011000000000000<br>1110000110110000000000000100100000100000000000
IR 58614-B-B-8-2	0000000000000101011110000000111000100111110100000100000<br>111011011000000000000011001001000000000110000100
KECHENGNUO NO. 4	0000000000000101000010001000000001110000000110000000100000<br>0001001000000000000000100010110000000100000000100
4484	1111000000000101000000000100000000111000000011000000100000<br>1100000110000000000000011000010010100000000001010
YOU-I B	0000000000110010010000010111000011010000001100110000000000<br>0011100011000000000000110010100001100000001110
CHUNJIANGZAO NO. 1	1111111111001000101111100000111100001111100111111111111<br>0001011000110111101100001100111101111111001001
Egyptian Wild Type	0000000000111100110000010000000000001000000000000000100010<br>11000001111110000000001001000000000001111001110
C.B. II	0000010000101100011101101000111000110000110100100110000<br>0010100000110000001010110010010000000001011110

Supplementary table7. Fingerprint coding of 284 varieties of *Sus scrofa*

Name	Fingerprint Encoding
ssc.s1	20002002002000100000001200222001000222202202000000002222000<br>002000111100010000200002000200002000000011
ssc.s2	00000000000000002000000000000020200000000002000000000000000<br>0001100000010020000102000000020000000000020
ssc.s3	00000020020001002000020000000000000202000020200000000000200<br>00012002000100000002020000000000000000020000
ssc.s4	00000200020000002000000000000000001000000202002000000000200<br>0001200000010020000002002000020000000200000
ssc.s5	00200020000000200000000000000000000102000000100002000000000<br>0001200000010010003002002000300000002000000
ssc.s6	00000000000002002000020000000000020000000000000000000200000000<br>000120000001002000020200000000000000000000
ssc.s7	00000200000000000001000000000000000000010000000000022000000000<br>0002100200210220000001002000200000000220010
ssc.s8	00000020020000000202002000000000000000200000000002000000000<br>0002100002210002000201002000000000000220020
ssc.s9	0020022000000000000200000000000000000200000000020020022200200<br>0002120000210020000002022000000000000200000
ssc.s10	00000000010000002000000000000000000202000000000000000000000000<br>000112000021000200000200100000000000200000

Name	Fingerprint Encoding
ssc.s11	0000000000000000200000000000000020200000000000000000220000<br>000110000201000200000200100000000000220000
ssc.s12	0000022000000200202000000000002000002000000000002020000000<br>000220020001000200000002200002000000000020
ssc.s13	0020002001000200100000000000001022002000000200000020000000<br>000110000201000000020200200000000000200020
ssc.s14	0020020000000200200002000000202000000000000000002000000200<br>000210020021022000000200200000000000000010
ssc.s15	0020002000000100002000000000000002002000000000000010000000<br>000112020001000200000200200000000001000000
ssc.s16	0020000000000200200002000000202020002000000000000020000200<br>000010000021000000000120200000000000000000
ssc.s17	0120000000001000200200000000000000000000002000002002000200<br>000012000001000000000000000002000002200000
ssc.s18	0220000220002002000200000000000000000000002000001002000100<br>000010001001000020000000000001000000200000
ssc.s19	0020000000002000000000000000000000000000002000001002000100<br>000010002001000000000020002001000002002200
ssc.s20	0220000000001000100000000000000000002000000200002002000100<br>000011000001000020000000000000000002000000
ssc.s21	2220000220001000200200000000000000000000002000000000000200<br>000010002001000000000000000002000000102000
ssc.s22	0002020000002000000000000000002002002000000012002000000000<br>000010000021000010000200000000000002000020
ssc.s23	0000000000000000000000000000022000000000000000000000000000<br>000300000001000000000000000000020032010300
ssc.s24	0000000100000000022000202222020020020020000020020122200000<br>002000211100122202210002000000100010000000
ssc.s25	2000020020222000001000001202002020020000000220020000000002<br>001000211120210201100020022000000000002210
ssc.s26	0000000000002220022010020000000000000000000000202000002200<br>200220002001010000000000000000000002000003
ssc.s27	2200000002000000000020000000222202000200002000220000000000<br>200210020021000020000000020000000000000020
ssc.s28	0100000000000000000000000000000002000000000000000000000200<br>000110000001020000000001002022000000000000
ssc.s29	0200000002000000000000000000000002000000000000000200000100<br>200010000000020000000002020002000000000000
ssc.s30	0200220000000220022202022222202002210000020102000100022020<br>022000011120210101222000002202000200000001
ssc.s31	2000200022022220022022021202001202210000200021000202012002<br>220000011110210001201002001200020000202001

Name	Fingerprint Encoding
ssc.s32	2002000022020222020202022201001000210002200001000200012000<br>000000011120212100002000002000000200202001
ssc.s33	2000020002002002020022201020001002020022200200200200122002<br>020000021122212201002020202220000000002021
ssc.s34	0001000220220002010002021220202202120020200201000100021000<br>020000011120210202212210200100202000200001
ssc.s35	2000220022020120012022021202002002020020100022000002100002<br>200200011110010202002000220220201000200021
ssc.s36	0000000000000000002000020000000000000002000000000000000000<br>100130000001020000000202000000200103000200
ssc.s37	00002000000000000000000020001000000000000000000020020002002<br>020110000201020000000000000000000001000200
ssc.s38	0200000000000000000000000000100000000002020000200200000000<br>200210000001000000000200200000000201002020
ssc.s39	0300000030000030300300000000030000030000303000303000003303<br>000003033001000000002000000000031030300300
ssc.s40	0200000000002000000000000000002000200000000000000200000000000<br>0000100000010200000002221000000002020000100
ssc.s41	010000000222200000000000000000200000000000000000002000000000<br>20001100000100000000000020020000000020000200
ssc.s42	0100000000000000000000000000020000002000200000000000000000<br>0000120000010000000000002002002002020000200
ssc.s43	0200000000002000000000000000002000010000000000000020000000000<br>0000100020020000200000100000000000000000001
ssc.s44	00000000022200000000000000000000000000002000002022200000000<br>00001000000100000000000020020000000000020020
ssc.s45	0200000000000000000000000000200000200000020000000000000000<br>0000220000010000000002000000000002020000100
ssc.s46	0000000002200000000000000000000001000000200000020000000000<br>00021200000100000000000020000000000000020001
ssc.s47	02000000012200000000000000000000000000000000000022000000000<br>00001100000100000000100000000000000020020200
ssc.s48	00000000002200000000200000000000002020000000000001020000000<br>00021000000100001000000000000000000022000200
ssc.s49	000020002000000000002000000001000000000000100000002000000000<br>00201000000102002000000000000000020000000220
ssc.s50	000002200022000000000000202022000220022002200000000000000000<br>0000100000010000000002000200000000002000220
ssc.s51	002000000000000000000000000000000000000000000000000020000000<br>0202100000010000200000000000000000000000000
ssc.s52	022002000000000000000000200000000000000020020200000000000000<br>0002100010000000000020000000000000000020001000

Name	Fingerprint Encoding
ssc.s53	00000000000000000000000000002000000200000000000000000000<br>2000200000010000000000200000000000002200000
ssc.s54	00000020022201000000020000002020200020000000002200000000100<br>0001100011010220000002102000200000000200200
ssc.s55	00000020020002002000000000000200020102000000000200000000000<br>0000120000010020000102002000000000022220000
ssc.s56	00000220000002002000010000000000100000000000000002000000200<br>0001110000010022000200202000020000002200010
ssc.s57	00000010000002002000000000000002000202000000200220000000000<br>0001110000210020000202202000200000000200000
ssc.s58	00200200000002002000020000002000200000000000000202020000200<br>0000200000201000000010022200002000000000000
ssc.s59	00002222201020020022020020010020000200000020002001010220000<br>0010002111000120022020220020222022000110001
ssc.s60	00000220000002000020000000000000120000000000000000000000000<br>0002100000210000000200011000020000000200020
ssc.s61	00000000020002002000020000000002000200000000000000000000000<br>0000100000010022000000202000020000000000013
ssc.s62	00200000000002000000000000000000000000020000000000000000200<br>0001000202010022000000202000000000002100000
ssc.s63	00000220000000000000000000003000002000000000000200200000000<br>0000000000010020000202000000200000001100003
ssc.s64	00021002022100000002000100202001000211020102222000002222020<br>0220001111000100200020022000022110000200001
ssc.s65	00020002021000002000022100222002000222000200022000000000002<br>0100001111000102020000000000202022000020001
ssc.s66	00000000000030000000000000300000000100000000000000000000000<br>3000100000030000000000000030000000300000000
ssc.s67	02000000010000000000000000000020000000000000000000000000000<br>0001100000200000000000100010000002000000001
ssc.s68	00200000000200222212220000020000020222022002020020000002000<br>0000002211202102002000020000200112000000001
ssc.s69	00020000000222002022002022022000200020022000202200002222000<br>0000001111202102001000010000200220020000001
ssc.s70	00202002002220022021000000200001202022222000202002000002010<br>0020000002210110000100021000020222202000001
ssc.s71	20020002000200000002022020002200200022212220220000000202002<br>0020000211120010002200002020210022000022001
ssc.s72	00002200100200020000000220022202000000000220000000000222000 1<br>0120002111000100011000002221010200200100 11
ssc.s73	00020100001000220002220020020201000202002000220000001012001<br>0120000111000102021000000020020000000010021

Name	Fingerprint Encoding
ssc.s74	1002020000000000002210002022100020011000020220200022100020<br>021000101100210002000000200000200000010001
ssc.s75	2000002020220020203220020002000200020120220020200000012000<br>001000121200010000020000200200220020020001
ssc.s76	0002000020100000101020220022100000012200220020200222100000<br>001000101100210000000002000202000200000021
ssc.s77	02000000000022000200000000000020002000021000000001000000000<br>200002000001000000000000002000000220000001
ssc.s78	000000000020210000000000000000002022000022000000002000000000<br>100020000111010000000200010000020000000200
ssc.s79	020022000020210000000000000000002200200000000000002020000002<br>100001002001200020002000000000020200000001
ssc.s80	022000000000200000000000000000002200020000200000002200000000<br>2000200000010000000000000020000002020000000
ssc.s81	000000000000010000000000200000020000000001000000022000000000<br>100020000111010000000200020000002020000020
ssc.s82	002000000000100000020000000000000020000021000000001000000002<br>00002000000012200200102000200000000002000001
ssc.s83	002000000000200000020000200000000000000000200000022000000002<br>200020022001200020000020020000000000000000
ssc.s84	000000000000010002000000200000000000000000200000001000000002<br>200000000111010000020220010000200020000000
ssc.s85	00100010000000000002000000000010000010000001002000000000000<br>020210000000100030010000000000000000001000001
ssc.s86	2000022200220020202020022202202222000022222200020200220022<br>02200002110022020222020020002022202000022021
ssc.s87	020033000330000000001330000000003000030000000003000003000003<br>000010003301000000030000000303300000030300
ssc.s88	010020100000000000001000000020200002022000000000000000002200<br>0000100000020000202000000200000002000000020
ssc.s89	00000000300000000000000030030000023300303000000000030001000<br>0000330030010000000000030000003300300000100
ssc.s90	000000000000000200002000000000020020020000020002000000002010<br>00001000000102000000010000000000000000000100
ssc.s91	020000000000000000002000000000000000001000000000000000000200<br>0000120000000000000002200000000020022000000
ssc.s92	000000220000000000001000001010200020020000000000000000000000<br>000000000000000000200000000000000000000000
ssc.s93	20021000022020000121000000000200020020020200202000102000002<br>002000011100212002102000202000021000000201
ssc.s94	2000110022022000020101020202200000122002100221000200002000<br>002000011100011001100020000022022000220201

Name	Fingerprint Encoding
ssc.s95	0002201101201020200020020100020202020002000002120200222020<br>020000211200210001200012020220222200020001
ssc.s96	1000200000021000211022001000020202200022021200000202122002<br>012000211100210222200000000222001000020001
ssc.s97	2001000220000200000102222022212000220010000202200212222010<br>100000021100212001020001002011000100210001
ssc.s98	0000200022010022012202010020221102200020000012020212222010<br>000002021100210001220002222122000100212001
ssc.s99	0001000202000220020201022000220000200020000001200022022010<br>100002011100110001000000222220002000022001
ssc.s100	0200002000220000010201010222102201020022000012000212221012<br>100001011100210002020002002220002000012011
ssc.s101	0002000022000210100201012202020100100220000002000210021010<br>100002011120212002020000202222002200210001
ssc.s102	0200002000000000000020000000002100200002000000000200000000<br>000022000000222002000020012000000001000020203
ssc.s103	0000000000000003000003000000000000030000030030000000000000<br>000010000303300000000303330030000002030000
ssc.s104	0000000000200000000000000030000000100200003000000000000000<br>300010000003030003000000000000000000003000001
ssc.s105	00020102020000022020022222220000000000002020201002000020000<br>022000010001010000200210000220201000000001
ssc.s106	2002220200000002002002010201000202020222012000002000020000<br>002000212201010000002010002220002000000001
ssc.s107	2201010200000222000201220020000000020200210002002200020222<br>022020012202010000000102000121100000020001
ssc.s108	20200200000000022000022002220000000000000012000000100020000<br>022000111100210001000100000111100000010001
ssc.s109	1202200202000010222002000002000000220200212002002000110001<br>011000222202210002000220020200220000000201
ssc.s110	0000001000000002000000000000020000000000000101000000000000<br>000110000000100000000000000000000000022000000
ssc.s111	0000002000000002000000000000020000000000000200002000000000<br>000110000000100000000000000000000000022000000
ssc.s112	0003010202010000000000012002020000000100000300100000000010000<br>013000011100212002232000000000000000000000201
ssc.s113	2001010022002022120000120210000020010200000122020002220002<br>012020021100012011020000002202222000000002
ssc.s114	00022000200000002200002101100000000010200210220200002222001<br>010000200002012000222000002120222000000001
ssc.s115	0000020100002002210020220220000020020220010122020002220020<br>012000011100011022220000000200202000000001

Name	Fingerprint Encoding
ssc.s116	0002000200002020100000120000000002000100010022212020222002 1<br>010000211100212122120000000002002200000000 1
ssc.s117	000220010000000020000021011010000000220000002022000022000 12<br>0110000111000101001020000000200002000000001
ssc.s118	00022202000000201202001202220000000102000202020000022200 22<br>0120000111000122222000000000200021000000001
ssc.s119	00000001000003201302001201102300000103000000130000000012011<br>0110000111000130200210000002000100000000 01
ssc.s120	000000010000000011000011022020300001000001002200000110000 0<br>0110000111000102003103000302010030000000 01
ssc.s121	0000020000002100000020020000000000200200002001000000000000 0<br>0000100000002000000000200220000000020000020 1
ssc.s122	000033030000030000030000100000000000003000000000000030000300<br>030010030001000010000300010000000003000000 0
ssc.s123	00022002002100220000220120202001200002022000202002202000 12<br>01200011110011000200000000002220000000200001
ssc.s124	000222000002002200102202201000022000000100000002020201000 2<br>2222222222002100000220001222222220002200201
ssc.s125	000220000001200000202102102020012002000200000002000202002 1<br>0101001111002202222020000100112000002002201
ssc.s126	100200022200002222021200220002022000001000000020020200202 0<br>0010000111000120002020120002000200002122 01
ssc.s127	0000020000220000000000000000000000000000000000000000000000200<br>00021200000100022000000000000000000021200200
ssc.s128	00000220000000000000000000200020000000000200002000000000000 22<br>0000200000002000000000020000030322000200000 0
ssc.s129	020000000000000200202002000001220000000000000000000000000002200<br>000230000000000000000000000000000020200200 0
ssc.s130	02200000000000220020220220000000000220000000000000000200000000200<br>002210002002000000000000000000000200000000 0
ssc.s131	0200000000000000000200022220000200022000000000002002000000002200<br>0002100020020000000000000200000000023000200
ssc.s132	01200000000000000000002022200302020120000000200000030003000 0<br>20001000022102000000000000000000000000002200
ssc.s133	0002000000000200000000220000020020120000020001022200000000 0<br>0002100020020000200000000020000000022002000
ssc.s134	00000030000000000020010020000020002200000000002002000000000 0<br>00021000100100000000202000200000000010002 01
ssc.s135	0020000000000000000000220000000000002202000000000000000000002000<br>2020100002220000000000000000000000120000020 0
ssc.s136	000200000000020000000220020000000201200000230000000000000000 0<br>000220002000000002000000000200000000020000200

Name	Fingerprint Encoding
ssc.s137	0200000000022100020010020000202002202000000000200000002000<br>200010002002000000020000020000000002000000
ssc.s138	0220000000000000000020000000002000200000200020001000000200<br>200210002002020010010000000000002000302020
ssc.s139	0220000000000120000020200000000001202000000020200000000100<br>000122002200000000000000200020020002000020
ssc.s140	0201002022202000221021000022002020022202002001000220200010<br>021000011100212001202002022220222000002001
ssc.s141	0002002222010000100002200000000222000200201101020220020020<br>002000111110210001201000000000201100002001
ssc.s142	0002002020020002010020200002002000022002000021000002200020<br>011000211120210001200002020000200200002001
ssc.s143	0000000000000000000000000000000000000000000000000000000200<br>000212000001000000000000000000000122000000
ssc.s144	0000000020000000001000000000000020000000000000000011000002<br>001000011110111100000000010000000100000100
ssc.s145	2000000020000000202000000000000020000010002000000200202001<br>000000021100010000202022020010102200222020
ssc.s146	2002022200222000022220000220000020000001220000000200222002<br>020000011120010010202020020000202000020021
ssc.s147	0000000000202200000002000000000200010000220000000220000000<br>100000020001022000020000000002000020000001
ssc.s148	0000202200000000202020000000020000022000200000000200000000000<br>000012002000000010002000000000002000200200
ssc.s149	0200100000220002000000000202000001020000000000000000002000<br>000010000002020010000000020000010022002000
ssc.s150	0000100200002002000200000202000002200200200020220000002000<br>000010002000000010000000002000001000200002 0
ssc.s151	0200102000220000000200000001000001000000000020200000002000<br>200220020002020010020000000000010021002000
ssc.s152	0200000000000002000000020101001002000220000000100000000200<br>000010000001200202000200020000020000000000
ssc.s153	0000200000000000000000000000001000200020020000020000000001100<br>000210000001000000020100000000022000000000
ssc.s154	0000200000000202000000000000000000000202012000101000000000200<br>200010000002000000000000000000202022000020
ssc.s155	0000200000000000000000000201010000000102220030103000001000<br>000010000002000000000100000000000010000000
ssc.s156	0000000000000002000000000000001200000020000000001300200021 00<br>000010000001000000000200000000010000000000
ssc.s157	0000000000000002000000000201020001200022000020000000000000<br>000010000002200000000200020002200010000000

Name	Fingerprint Encoding
ssc.s158	010000000000020000002000200002200000000200000000000000000<br>20000002000201000000000000000001000200000001
ssc.s159	000000000000000000020000000202100002000100000001000000000200<br>20001000200102000000020002000000200200000000
ssc.s160	000000002000220000020020000002200000000012000000000000000<br>00000200002200000000000000000020000220020001
ssc.s161	000000000202000000000000000000200002000222000000200000000<br>20002000200000000000002220000020002200020001
ssc.s162	000000000000220000000200020000020002000020000000010022000000<br>00000000000101000000201000000200002000000001
ssc.s163	020200000000220000000000020000020001000010200000000000000000<br>20000002200202000000220002200000000020000201
ssc.s164	000010000200200202002002020000200022020000002020200000000000<br>00001000000100002000000000000000020001002000
ssc.s165	000020200011000102022000010202000100000000000020000000000000<br>00022002000202001000000000000000100220000000
ssc.s166	000020020222000102022000000000000020020002000001002000000000<br>00021000200200200000000000000002020000000000
ssc.s167	200000000000020000012000000010000000000000000000000000000200<br>02001002000010000100000002020000022200201
ssc.s168	000000000000000000120000200200000000000000000000000000000000<br>22201001000102020200000000000200002200000000
ssc.s169	200002000000002100202000022000000000000200221202020010000 1<br>02000001110001200100000200102022002010020
ssc.s170	000000020000001202200000102000000000000220000200202102200 01<br>02200001110001000220200102100002002002001
ssc.s171	000001000010002000201020211020020000000002212000022000000 1<br>01000001110001000100000100220010002022002 0
ssc.s172	200001000000001100000000000202000000000020002222000100200021<br>02000001110001000110000000122022002000001 0
ssc.s173	200002212020000000200020220020000000000202222000022000000 1<br>01000001110001000120000202100020000022002 0
ssc.s174	000001022000000200200000212010000000000000222100200100000001<br>02000001110001000100000000202000200010001 0
ssc.s175	000002202000000200001000222200020000201001000000000000000 01<br>01000001112001000120100002101020000022002 0
ssc.s176	200002000000002000202000022000022000000000200000001000200 01<br>01200001110001000110000200122001000000200 1
ssc.s177	000201000000000000020202000220002000020200220220001002100 01<br>02000001110001000010000200122022200010200 1
ssc.s178	200002000000002200202000222000022000000000222222202202000 01<br>02000001110001200110000200201021000022022 0

Name	Fingerprint Encoding
ssc.s179	0000010000100011000000010000000000000001200000001000000000001<br>02000001110001101000100000100010100000000
ssc.s180	0020000000000000000000000000200000000000000020002000000100<br>00001000000100002000000000000000000022000200
ssc.s181	00000000000000000000000000000000000000000020000000000100<br>00021100000100002000000000000000000021000000
ssc.s182	0020000000000000000000000000100000000000000000000000000000<br>00001200000100000000000000000000000001000200
ssc.s183	0010000000000000000000000000200000000000000000000000000200<br>00001000000100000000000000000000000002000100
ssc.s184	00000000000000000000000000000000000000000020000000000200<br>00001000000100002000000000000000000011000200
ssc.s185	0003000000000000000030003000000000000300030000000000020003000<br>0000100000013000000001000003000000000000000
ssc.s186	000000000000000000000000000020000000000000100000202000000<br>0000100000010000000000200000000000000000000
ssc.s187	000000000000000200021000000002100200000000002022000100000001<br>02200001112001120000102000000000001200001020
ssc.s188	0000000220100011022100012020200220000220000220010222202022<br>00200222110001000200000200222020002002 0000
ssc.s189	0000002000000000010010000020000010000222020000000120020000<br>00100201110001300200202102202223000001221
ssc.s190	2002200020000000001001002020000020000002000200220020022202002<br>00200021112001000022002200202020020022 0001
ssc.s191	1000000000000020102000002100000000000000102000000000102 30022<br>00200001120001000110200200201000221020 0200
ssc.s192	0000000002000020000000002200000001000201100100202002 0120022<br>00200001112001100210100100102002001000 2001
ssc.s193	00000202220000000220000012200002000002110022000202001 10021<br>00000001110001200220200000001002220020 1021
ssc.s194	0020000000000000000000000000100000000000000000000000000200<br>00001200000100000000000000000000000010000100
ssc.s195	00200000000000000000000000000000000000000010000000000000100<br>00001000000100000000000000000000000010000100
ssc.s196	002000002000000000000000000000000000000000000000000000000100<br>00001200000100000000000000000000000002000000
ssc.s197	0000000000000000000000000002000000000000000000001000000000<br>00001200020100002020020000000000000211000200
ssc.s198	00000000000000000000000000000000000000100000202000200000000000<br>00001100000100000000000000000000000002002000
ssc.s199	00000000000000000000000000000000000000200000102000100000000000<br>00001100000100000000000000000000000201000000

Name	Fingerprint Encoding
ssc.s200	0000000000000000000000020000000000000000001000000000000000<br>000010000001000000000000000000000200000000
ssc.s201	0000000000000000000000000000020000000000000000000000000000<br>000000000001000000000200000000000022000100
ssc.s202	0000000000000000000000000000000000000000001000002000000000<br>000020000001000000000000000000000000000200
ssc.s203	0020000000000000000000000000000000000000000000000200000000<br>000011000001000000000000000000000202001200
ssc.s204	2001002000022001002002010020000202012022020200000200021022<br>011000011120010000102020000002210200020102
ssc.s205	0002020202220202020001220000020200200000220002020020100020<br>020020022220020000000000100010201200002001
ssc.s206	2200022022021200002022222000202000022002120200000200002020<br>002000221120010201002012020202000000201221
ssc.s207	2000222222022222020200222002000202200020200202020120220020<br>000200011120210001020020000222000200121021
ssc.s208	2001020011022002020201002220202000200000020000010002102000<br>221021001200012201020000222212200000002021
ssc.s209	1002002202002000002002100002020002220020200000201002222200<br>000200021102010002000000200222200000000021
ssc.s210	0000102221012012210102222022002202020020020001220211202000<br>202000111100010201000000221222020000022011
ssc.s211	0200202000010202010100000101001100022200200202020220220020<br>011000011100110011000000000110021000022201
ssc.s212	0202200002010200000100100002002202020200220202200200000020<br>022000011120210101000000020100011000022001
ssc.s213	2000022222220000202022222202002220020022222202020202220020<br>222000221120220202202022002002202200022021
ssc.s214	2000000222202020021000202200000020000222000002010202022000<br>021000221100010002010000220200020200002001
ssc.s215	2000222200220020002220022202202222022022222200020200020020<br>022000222200220202200022202002232000022022
ssc.s216	2000020200210022022220002022022212000012002000020220001022<br>021000011120010000200002002220220200000000
ssc.s217	0000000020020021220100000020220000020220020001010200000012<br>000000011110010000202002200120100000012200
ssc.s218	0000200010000000000000000000000020000000000000000001000012<br>001000111110111100002020010000000000000000
ssc.s219	0000100010000000000000000000000010000000000000000001000010<br>001000111110111100002000010000000000000000
ssc.s220	0000100010000000000000000000000020000000000000000001000010<br>001000111110111100002000010000000000000000

Name	Fingerprint Encoding
ssc.s221	0000200010000000000000000000000001000000000000000000001000010<br>03100011111011111000020200100000000000000000
ssc.s222	0000100010000000000000000000000001000000000000000000001000010<br>00100011111011111000010200100000000000000000
ssc.s223	0000200010000000000000000000000001000000000000000000001000010<br>00100011111011111000010200100000000000000000
ssc.s224	0000200010000000000000000000000002000000000000000000001000010<br>00100011111011111000010000100000000000000000
ssc.s225	0200000020002000000000020000002200000000220000000220000000<br>100200020002012000000000020000000020000001
ssc.s226	0000000000200000000000000000000200020000000000000000000200000<br>200000020001020000002000000000000000000001
ssc.s227	2200002002220020200002200000000000000200002002010020000020<br>100111111200020010000020202000000200120000
ssc.s228	2022000000222000000200002000202000100002200000012100002200000<br>200222211200000200010000101000000000200001
ssc.s229	1122000000000000002000020000002022000020000000000000202200000<br>200320222002002200010200202000002200000020
ssc.s230	2022000002000000002000020000002022000022000000000000200200000<br>200020022202002200020200202000002200000020
ssc.s231	2220000002000000220000200000020020000220000000020020220000<br>100200111002002200020200202000002200000000
ssc.s232	0022200000222000000000022000202000200201200100022000000000200<br>200222111000020200000000100000200000000001
ssc.s233	1000002001120020000200000020200000000020000000010000000000<br>010111111100000020000000100000000200220000
ssc.s234	0100001001120020000201000010200100000210020000221002022002<br>220111111200020010000000002000000000120000
ssc.s235	0222002001200010220201000020100000000020000000221002022002<br>010122111200020010000000202000002100200000
ssc.s236	0120000001220000020202000010000200000200020000110020000000<br>220111111100000010000020201000002200120000
ssc.s237	2220002001120020220001200020100200000020000000111002022002<br>220122111200000010000020202000000100200001
ssc.s238	2211000000200000000200002000000202200002200020000200000000000<br>200000211202000200020200202000002200000020
ssc.s239	1211000000000002021000010002022001000012002200222000000000020<br>100000110001000000002000010200000000000000001
ssc.s240	0212000000022000002200002000000000220000200020000000000000000010<br>100211111200020020020200102000002000200001
ssc.s241	0220000100002000200000000000000000000001000000000000000002000100<br>000011000001000010000020000002000000101000

Name	Fingerprint Encoding
ssc.s242	0120000000000200000000000000000000000000000000000002000100<br>0000120000010000100000000000001000000102200
ssc.s243	0220000200002000000000000000000000000000000220000200200020 0<br>0000100020010000000000100000010000002200000
ssc.s244	0120000100001000000000000000000000002000000100000000000100<br>0000110000010000200000200020000000000100000
ssc.s245	0110000220002000000000000000000000002000002000002000000100<br>0000100020010000000000000002001000000200000
ssc.s246	0220000000000200000000000000000000000000000220000000200020 0<br>0000110000010000200000200000020000002202000
ssc.s247	0020000200000000000000000000000000002000002000002000000100<br>0000100020010000000000200020010000002202000

Supplementary table8. Two-dimensional code of *Manihot esculenta* Crantz fingerprint

Name	QR Code	Name	QR Code
SC8		xinxuan048	
C4		FB-6	
KU50		NZ199	
SC5		SC6	
SC205		17Q	
